# Proteinaceous Hydrogels for Bioengineering Advanced 3D Tumor Models

**DOI:** 10.1002/advs.202003129

**Published:** 2021-01-04

**Authors:** Barbara Blanco‐Fernandez, Vítor M. Gaspar, Elisabeth Engel, João F. Mano

**Affiliations:** ^1^ Department of Chemistry, CICECO – Aveiro Institute of Materials, University of Aveiro Campus Universitário de Santiago Aveiro 3810‐193 Portugal; ^2^ Institute for Bioengineering of Catalonia (IBEC) The Barcelona Institute of Science and Technology Baldiri Reixac 10–12 Barcelona 08028 Spain; ^3^ Materials Science and Metallurgical Engineering Polytechnical University of Catalonia (UPC) Eduard Maristany 16 Barcelona 08019 Spain; ^4^ CIBER en Bioingeniería Biomateriales y Nanomedicina CIBER‐BBN Madrid 28029 Spain

**Keywords:** 3D in vitro models, cancers, hydrogels, peptides, proteins

## Abstract

The establishment of tumor microenvironment using biomimetic in vitro models that recapitulate key tumor hallmarks including the tumor supporting extracellular matrix (ECM) is in high demand for accelerating the discovery and preclinical validation of more effective anticancer therapeutics. To date, ECM‐mimetic hydrogels have been widely explored for 3D in vitro disease modeling owing to their bioactive properties that can be further adapted to the biochemical and biophysical properties of native tumors. Gathering on this momentum, herein the current landscape of intrinsically bioactive protein and peptide hydrogels that have been employed for 3D tumor modeling are discussed. Initially, the importance of recreating such microenvironment and the main considerations for generating ECM‐mimetic 3D hydrogel in vitro tumor models are showcased. A comprehensive discussion focusing protein, peptide, or hybrid ECM‐mimetic platforms employed for modeling cancer cells/stroma cross‐talk and for the preclinical evaluation of candidate anticancer therapies is also provided. Further development of tumor‐tunable, proteinaceous or peptide 3D microtesting platforms with microenvironment‐specific biophysical and biomolecular cues will contribute to better mimic the in vivo scenario, and improve the predictability of preclinical screening of generalized or personalized therapeutics.

## Graphical abstract

The most recent and relevant protein and peptide‐based hydrogels used in 3D in vitro tumor modeling and therapeutics screening are discussed herein, outlining the importance of employing biomaterials for mimicking the 3D tumor microenvironment (TME) in vitro. The microengineered approaches used to recreate the TME and the future challenges that should be addressed to improve their predictability are also discussed.

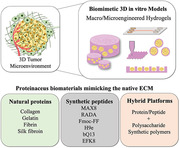

## 1 Introduction

Cancer is currently the second leading cause of death worldwide, requiring that pharmaceutical and biotechnological industries invest a significant amount of resources and effort every year in the discovery and development of more promising therapeutics. However, the attrition rates of most candidate therapeutics are remarkably high, with only few new molecules being approved by regulatory agencies, due to toxicity or efficacy issues.^[^ ^1^, ^2^ ^]^ These high failure rates are associated with tumors inherent complexity and heterogeneity, to the lack of sufficient knowledge on cancer pathophysiology and to the absence of tumor biomimetic preclinical screening models. The latter is vital to understand how each individual parameter affects disease progression and assist in new drug targets discovery.^[^ ^3^ ^]^ Moreover, more accurate and predictive preclinical models could assist on improving drug research and development efficiency by giving a robust therapeutics performance analysis that could be more translatable to the results obtained in vivo.

Nowadays, 2D in vitro and in vivo animal models remain the gold standard for screening the efficacy of candidate anticancer therapeutics, and they are still in use to also study cancer physiology. Preclinical in vivo models enable researchers to evaluate tumor progression and screen new molecular entities in a multiorgan and biologically relevant environment.^[^ ^4^ ^]^ Nevertheless, there are important differences between rodent physiology and humans.^[^ ^5^ ^]^ The alterations in the species genome provoke small biochemical, cellular, and anatomical variations. These dissimilarities are responsible for organs and biosystems functionalities (immune system activity, liver metabolism, kidney excretion) and anatomical (organs size or composition) disparities. In addition, when human cancer cells are studied on animal models, immune‐compromised animals are generally required to minimize cell rejection, which might reduce the reliability of these models.^[^ ^6^ ^]^ The CRISPR technology has recently enabled the use of animal tumor models with human cancer cells,^[^ ^7^ ^]^ and in the last years mice with humanized immune systems have also been established as human tumor models.^[^ ^8^ ^]^ Nonetheless, such models are costly, exhibit a low data throughput and are unable to fully recapitulate key hallmarks of human tumors. These intrinsic limitations are responsible for a high failure rate of candidate therapeutics when evaluated at clinical trials level despite the positive results generally obtained in preclinical studies.^[^ ^5^, ^9^ ^]^ On the other hand, regulatory agencies and governmental institutions are also actively seeking to implement a reduction in laboratory animal research.

On the other hand, 2D in vitro models, are extensively used for drug screening and cancer modeling, especially for high throughput screening (HTS) during anticancer therapies preclinical in vitro screening stages. 2D models, comprising primary human cells or commercially available cell lines are cultured in a monolayer, are easy to handle, reproducible and cost‐effective. However, the lack of a 3D environment can influence the predictive conclusions retrieved from the use of these models. Such is particularly evidenced by different results in drug efficiency, cellular morphology, gene, and protein expression when cells are cultured in 2D flat monolayers versus their 3D counterparts.^[^ ^10^, ^11^, ^12^, ^13^ ^]^ Indeed, these models cannot recapitulate the actual physiological environment to substitute completely the animal models,^[^ ^14^ ^]^ although their use contributes to the reduction of animal testing.

Apart for the 3D culturing conditions, another of the key reasons why preclinical models fail is that, in general, they recapitulate tumors solely as a mass of cancer cells. However, the tumor microenvironment (TME) plays an important role in tumor progression and treatment efficacy.^[^ ^15^, ^16^, ^17^, ^18^, ^19^, ^20^, ^21^ ^]^ Hence, the TME needs to be also recapitulated in preclinical stages to guarantee the success of candidate therapeutics in clinical trials. Recapitulating the TME in vitro enables the identification of new biological targets and may also facilitate the discovery of more effective therapeutics/therapeutics combinations for cancer treatment.^[^ ^22^ ^]^

The purpose of this review is to discuss the current landscape of protein and peptide‐based single or hybrid hydrogels as TME mimetic scaffolds for developing biomimetic in vitro cancer models that recapitulate key tumor hallmarks. Their biocompatibility, similarity with the tumor extracellular matrix (ECM), the presence of motifs for cell adhesion and biodegradation and milder jellification process compatible with cell encapsulation are some of the main reasons why they are still highly used in cancer modeling. We discuss the importance of in vitro 3D tumor models for the recapitulation of the TME in comparison with 2D and in vivo models. Moreover, we provide a comprehensive discussion regarding protein/peptide‐based hydrogels or their combination with other polymers and also highlight the microtechnological approaches employed for their processing and combination with cells toward obtaining living 3D microtissues (MTs). The materialization of more physio‐mimetic proteinaceous hydrogel‐based platforms for 3D in vitro tumor model's generation is envisioned to boost their mimicry of the in vivo scenario, while also providing a technological framework that allows researchers to screen for candidate therapeutics in a high‐throughput/high‐content mode.

## 2 3D In Vitro Models to Recapitulate the TME

**Figure 1:**
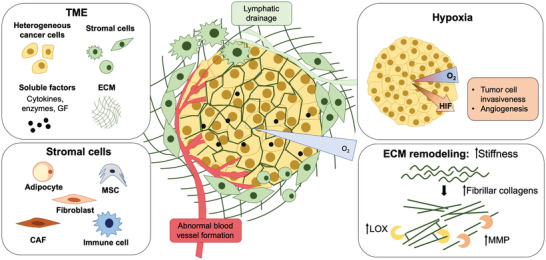
Schematics of the TME and its key elements. Tumors are formed by a heterogeneous population of cancer cells, stromal cells, ECM, and soluble factors (e.g., cytokines, growth factors). The ECM is a complex 3D nanofibrous network of proteins that provides cell support and regulates several cellular functions.^[^ ^26^, ^27^ ^]^ As the 3D tumor mass progresses, there is an abnormal ECM production and remodeling, increasing the production and crosslinking of some types of collagen by lysyl oxidase (LOX), upregulation of matrix metalloproteinases (MMP), and subsequently, increasing the stiffness inside and around the tumor.^[^ ^28^, ^29^ ^]^ Stromal cell populations include fibroblasts, immune system cells, endothelial cells, mesenchymal/stromal stem cells (MSCs) and adipocytes. As the tumor grows, the impaired blood circulation generates and hypoxic environment, promoting the secretion of hypoxia‐inducible factors (HIFs), increasing cancer cells invasiveness and the formation of abnormal blood vessels.^[^ ^15^ ^]^ Among stromal cells, tumor‐associated macrophages (TAMs) and cancer‐associated fibroblasts (CAFs) arise as one of the most important elements. TAMs are responsible for establishing an immunosuppressive TME^[^ ^19^ ^]^ and CAFs crosstalk with the cancer cells promotes tumor progression, metastasis and drug resistance.^[^ ^15^, ^17^, ^18^, ^30^ ^]^ The dynamic exchange of cytokines/growth factors (GF) in the TME can also stimulate or inhibit several signaling pathways of cancer cells for survival, proliferation, migration, polarity, or differentiation.^[^ ^15^, ^31^, ^32^ ^]^

In recent years, significant efforts have been focused on creating 3D in vitro models that better recapitulate key features of the TME, to overcome the limitations of 2D in vitro models and to bridge the in vitro/in vivo correlation gap. Tumors are formed by an heterogeneous population of cancer cells, as well as of resident and infiltrating stromal cells, the supporting ECM, and soluble factors (e.g., cytokines, growth factors (GF), etc.), all together forming the so termed TME (**Figure** **1**).^[^ ^18^, ^23^ ^]^ In the TME, cancer cells establish a dynamic crosstalk with all the components of the TME, either via physical, metabolic or biochemical cues.^[^ ^15^ ^]^ Remarkably, cancer cells modify the TME to be more permissive toward supporting tumor growth and metastasis.^[^ ^24^ ^]^ The TME plays also an important effect in chemotherapy/radiotherapy therapeutic outcome.^[^ ^16^, ^25^ ^]^

### 2.1 Mimicking the 3D Tumor Environment

Cells living in 3D environment exhibit a completely different cytoskeletal arrangement, gene expression and metabolism than that observed in 2D flat cultures, better recapitulating the in vivo scenario.^[^ ^33^, ^34^ ^]^ Indeed, it is well known that the signaling cascades emitted by the TME and received by cancer cells (TME crosstalk) are fundamental for their phenotype and bioactivity.^[^ ^35^, ^36^ ^]^ In fact, in 3D models, cancer cells are closely interacting, but also establish interactions with other cell types and the materials mimicking the supporting tumor ECM. Indeed, the ECM is directly involved in the tumor progression, such as tumor stemness, epithelial–mesenchymal transition (EMT), metastasis or drug response, among others, representing up to 60% of the TME.^[^ ^37^, ^38^ ^]^ During the tumor progression course, there is a change in the ECM composition, like increase in some proteins such as fibrillar collagens, laminin or fibronectin, which lead to changes in the architecture and mechanical/physicochemical properties in comparison to the healthy counterpart.^[^ ^37^ ^]^ Moreover, ECM proteins have specific domains that enable cell adhesion through integrins,^[^ ^39^ ^]^ and these cell‐adhesion proteins play an important role in the tumor progression and invasiveness. The use of proteinaceous materials, or their hybrid combinations can provide the biochemical and biophysical cues necessary for cell proliferation, migration, and several other cellular processes,^[^ ^40^, ^41^ ^]^ which are essential for tumor progression. However, in 2D cultures, cells are only interacting though their junctions, as they are proliferating while being attached to a stiff polystyrene surface on one side and on the other contacting with cell media.^[^ ^42^ ^]^

In 3D solid tumors, oxygen and nutrients need to diffuse through the matrix and the dense cell mass, generating gradients that impact tumor progression and resistance to therapeutics.^[^ ^43^ ^]^ These gradients have been recapitulated in in vitro 3D tumor models,^[^ ^44^, ^45^ ^]^ but they cannot be mimicked in monolayer cultures, as they are exposed to uniform oxygenation and nutrition. Moreover, in 3D in vitro solid tumor models, cells can migrate in all directions, allowing to better modulate invasion and metastasis, an aspect which cannot be reproduced in 2D monolayers. Interestingly, in 3D models natural mechanisms of migration such as ameboid morphologies and lamellipodial protrusions can be observed, thus being closer to the in vivo scenario.^[^ ^46^ ^]^ It has also been observed that cells exhibit different gene expression patterns when cultured in 3D versus 2D, this can be occasioned by the signals coming from the TME and can play an important role in tumor progression.^[^ ^42^, ^47^ ^]^ Lastly, when cells are cultured in a 3D setting, there is a physical barrier for the permeation of therapeutics into deep tumor regions,^[^ ^48^ ^]^ and it is possible to mimic multidrug resistance (MDR) mechanisms.^[^ ^49^ ^]^ MDR is generally provoked by an upregulation of MDR‐associated proteins motivated by the onset of hypoxia, low nutrients and acidification in the solid tumor model in vitro. This behavior can be partially mimicked in 2D environments using commercial cell lines expressing MDRs, but differences in the expression of such MDRs can still been appreciated between 2D and 3D models,^[^ ^50^ ^]^ as the TME plays an important role in the establishment of MDR.^[^ ^51^ ^]^

### 2.2 3D Approaches for Cancer In Vitro Modeling

All the evidences described in the section above indicate that 3D models can recapitulate many characteristics of the TME, enabling the construction of more physiological relevant models, and their inclusion in the preclinical stage of clinical trials could be beneficial for evaluating the efficacy of new therapeutics. Particularly, researchers have mainly focused on developing two different testing models: i) scaffold‐free and ii) scaffold‐based platforms.^[^ ^52^ ^]^

In scaffold‐free platforms, cells are generally induced to aggregate into microsized 3D clusters (i.e., 3D spheroids). Such MTs can comprise cancer cells (homotypic spheroids), or include cocultured tumor and stromal cells (e.g., fibroblasts, mesenchymal/stromal stem cells, immune system cells or endothelial cells, etc.) (heterotypic spheroids).^[^ ^14^ ^]^ 3D spheroids can be obtained through cells culture in ultralow attachment surfaces, via the hanging drop technique, spinner flask bioreactors, or using superhydrophobic surfaces.^[^ ^53^, ^54^, ^55^ ^]^ 3D spheroids can recreate key features of solid tumors, including: de novo ECM deposition,^[^ ^56^, ^57^ ^]^ cell–cell and cell–ECM interactions,^[^ ^56^, ^57^ ^]^ grow kinetics,^[^ ^58^ ^]^ as well as nutrient, oxygen, and metabolite gradients,^[^ ^59^ ^]^ among others.

The oxygen deprivation in 3D spheroids core promotes hypoxia and the production of hypoxia inducible factors (HIF), potentiates the malignancy of the cancer cells (i.e., differentiation and metabolism) and can have an impact in anticancer drugs efficacy, similarly to what occurs in in vivo solid tumors.^[^ ^57^ ^]^ Since 3D tumor spheroids exhibit closer drug response and gene expression to that of in vivo tumors,^[^ ^60^ ^]^ they have been extensively used for HTS.^[^ ^14^ ^]^ Nevertheless, one of the main limitations of 3D spheroids, is the lack of preexisting ECM cues and the absence of an organotypic tumor bioarchitecture.

Recently, another interesting scaffold‐free approach consisting in tumor tissues has been developed.^[^ ^61^, ^62^ ^]^ These tissues are formed by the cell accumulation in well plates, by previously coating cells with a layer of fibronectin and gelatin. This technology enables the obtaining of clinically relevant fibrotic tumors, such as the ones observed in pancreatic ductal adenocarcinoma^[^ ^61^ ^]^ or even the construction of vascularized and lymph irrigated tissues to study the cancer cell invasion.^[^ ^62^ ^]^

On the other side, in scaffold‐based platforms, cells proliferate while anchored or encapsulated into/on top of a tumor ECM mimicking matrix. Such 3D scaffolds provide the mechanical and biochemical cues, as well as support cell proliferation.^[^ ^63^, ^64^ ^]^ 3D scaffolds have to date been fabricated from natural polymers (e.g., proteins, polysaccharides), from synthetic materials (e.g., peptides, synthetic polymers—polyethylenglycol, polyvinylalcohol, polycaprolactone, polylactic acid, etc.), and from their hybrid combinations. The generation of scaffold‐based platforms for 3D cell culture can be performed in a top‐down or bottom‐up mode. In the latter, cells are encapsulated within the biomaterial matrix, with hydrogels and hydrogel bioinks being the most representative examples.^[^ ^65^, ^66^ ^]^ In top‐down approaches, cells are seeded on top of a preformed scaffold.^[^ ^63^ ^]^ Among them, fibers,^[^ ^67^, ^68^ ^]^ foams,^[^ ^69^ ^]^ porous/solid microparticles^[^ ^70^, ^71^, ^72^ ^]^ and decellularized cell‐derived and tissues/organs‐derived matrix scaffolds^[^ ^73^, ^74^ ^]^ have been widely used. The main limitation of these scaffolds is related to the necessity of cells infiltration into the structure, an important technical aspect, that generally leads to difficulties in guaranteeing a homogeneous cell distribution and infiltration throughout the whole structure. Some reports have also demonstrated that cells cannot recapitulate the 3D environment of solid tumors when cultured in this mode.^[^ ^63^, ^64^ ^]^ The requirements that artificial ECM scaffolds need to meet are variable, depending on the tumor type and progression state. Biocompatibility, biodegradability, stiffness, topography, architecture, porosity, or cell adhesion sequences are only few of the features that need to be considered.

## 3 Hydrogels and the Tumor Microenvironment ECM

In vivo the tumor ECM provides the biochemical and biomechanical cues that are essential in tumor progression, invasion and metastasis.^[^ ^75^ ^]^ ECM properties such as composition, stiffness/viscoelasticity, permeation to oxygen and nutrients, topography and 3D architecture not only influence tumor evolution, but also treatment efficacy.^[^ ^40^, ^41^ ^]^ In the TME, cancer and stromal cells remodel the ECM by de novo deposition/crosslinking and degradation, rendering it more permissive for tumor growth.^[^ ^76^ ^]^ This also plays a very important role in resistance to therapeutics as crosslinked, dense ECM poses a physical barrier to bioactive therapeutics passive diffusion.^[^ ^18^, ^77^, ^78^, ^79^ ^]^ The importance of the tumor ECM and its high heterogeneity have motivated the design of new biomimetic platforms that can recapitulate the complexity of the TME and offer more predictive potential on therapeutics efficacy or contribute for a better understanding of fundamental cancer cells biology.

**Figure 2:**
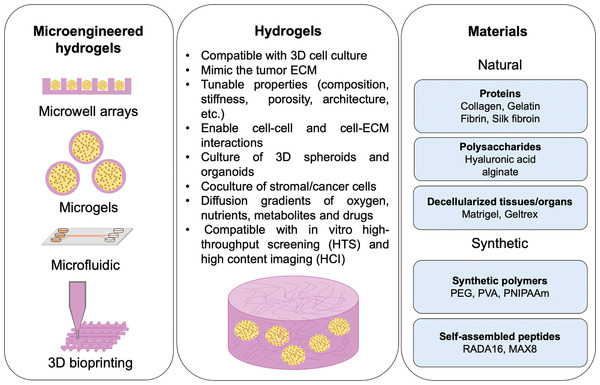
ECM‐mimetic hydrogel‐based platforms for establishing physiomimetic 3D in vitro tumor models.

Hydrogels have emerged as new supporting materials for 3D cell culture.^[^ ^65^, ^80^, ^81^, ^82^ ^]^ Hydrogels are 3D polymeric networks with high water content, resembling the bioactivity, viscoelasticity and mechanical properties of native tissues ECM.^[^ ^83^ ^]^ Hydrogels are particularly interesting for in vitro disease modeling due to their biochemical and biophysical tunability (**Figure** **2**).^[^ ^83^ ^]^ In fact, hydrogels architecture/porosity,^[^ ^84^ ^]^ shape, spatial distribution of cell adhesion motifs,^[^ ^85^ ^]^ mechanical cues,^[^ ^86^, ^87^ ^]^ and chemical composition can all be controlled to generate appropriate microenvironments for cancer cells proliferation and 3D microtumors formation.^[^ ^14^, ^81^, ^88^, ^89^, ^90^, ^91^, ^92^ ^]^ Moreover, the cellular arrangement inside hydrogel networks might influence drug response and cellular behavior.^[^ ^93^ ^]^ Monteiro et al. proved that osteosarcoma cells growing as a cellular agglomerated 3D spheroid in a Matrigel or gelatin‐methacrylamide (MA) microhydrogels have a higher drug resistance and invasiveness behavior than when cultured as randomly dispersed entities in typical cell‐laden hydrogels, highlighting the importance of modeling cellular aggregation (3D spheroids) in ECM‐mimetic hydrogels.^[^ ^93^ ^]^

Hydrogels can be generated under mild conditions compatible with cancer and stromal cells encapsulation.^[^ ^94^ ^]^ Also, their transparency enables the in vivo/in vitro analysis of encapsulated cells by using advanced microscopy techniques that allow high‐content imaging. Such approaches are critical for obtaining highly relevant data on key parameters including cell viability/proliferation, cell–cell adhesion molecules expression, necrotic core formation, or hypoxia onset.^[^ ^95^ ^]^

In addition to being used for 3D spheroids culture, ECM‐mimetic hydrogels are also suitable platforms for the culture of organoids. Organoids are 3D multicellular structures that replicate the genotype, phenotype, microarchitecture, and cell functions of tissues/organs.^[^ ^96^ ^]^ They are originated from stem cell populations which differentiate into different cells types and self‐organize in 3D, mimicking the structure of a tissue/organ following the silencing of key signaling pathways and administration of specific GF mixtures.^[^ ^96^ ^]^ Organoids are generally established in Matrigel hydrogels.^[^ ^97^ ^]^ However, its high inter‐batch variability has recently encouraged the use of collagen and polyethylene glycol (PEG) hydrogels as new alternatives for organoids culture.^[^ ^98^, ^99^ ^]^ Tumor organoids have shown to be a valuable platform for modeling human neoplasia, to identify new biomarkers and predict drug response in a patient‐personalized mode when patient derived organoids are employed.^[^ ^100^, ^101^, ^102^ ^]^ Although organoids recapitulate the architectural complexity and are more physiologically relevant, there are still important aspects of patients TME that are yet to be recapitulated.^[^ ^100^, ^101^ ^]^ Also, considering the high cost and complex culture conditions required for organoids establishment such models still require further optimizations so that they become broadly available. Nevertheless, they promise to be the next generation of 3D in vitro models for drug screening and fundamental tumor biology studies.

### 3.1 Microengineered Hydrogels for Tumor 3D Growth

Hydrogels can be processed via different microfabrication technologies, to recreate more accurate and sophisticated platforms that recapitulate key aspects of the TME.^[^ ^94^, ^103^ ^]^ The use of these advanced technologies, in comparison with standard macroscopic 3D hydrogels, enables a high control over hydrogels size/shape that may mimic that of the original tumor tissue/organ, and the establishment of the cell growth under dynamic conditions, as well as a control over the spatial distribution of stromal cells/vasculature. Such methodologies also allow to increase the reproducibility, lower costs, and are compatible with HTS.^[^ ^104^ ^]^

One promising example are the use of emerging 3D bioprinting technologies, such as extrusion‐based bioprinting which allow the fabrication of complex structures by sequential deposition of ECM mimetic biomaterials in stacked layers, where cells can be encapsulated within the bioink or seeded after the bioprinting process. This enables the fabrication of precise structures with spatial control,^[^ ^105^ ^]^ enabling the construction of anatomically relevant 3D tissues and organs,^[^ ^105^ ^]^ even in gravity defiant structures.^[^ ^106^ ^]^ This technology has implemented the knowledge from different disciplines, such as chemistry, materials, tissue engineering, or disease modeling,^[^ ^107^, ^108^, ^109^ ^]^ and it is compatible with high throughput production and microfluidic devices.

Another interesting approach is the combination of hydrogels with microfluidic devices. The combination of microfluidic technologies and biomedicine has evolved the field of disease modeling with the implementation of organ‐on‐a‐chip platforms. These tumor‐on‐a‐chip (TOC) devices can be tailored to mimic the most relevant characteristics of the TME, to study cancer progression and for HTS. These platforms recapitulate the microscale interactions in the TME, recreating the physiologic‐distances between components, being suitable to study cancer cells/stromal cells/ECM interactions.^[^ ^110^, ^111^ ^]^ Also, they can provide the establishment of physical/chemical gradients in the device, such as hypoxia, molecules concentration gradients, or shear stress, an approach that can be used to study how these conditions affect the tumor progression or chemotherapeutics resistance.^[^ ^112^ ^]^ They also allow the study of the effect produced by specific therapeutics, nanoparticles (NPs) or cells, angiogenesis,^[^ ^113^, ^114^ ^]^ or to study cancer cells extravasation or micrometastasis,^[^ ^115^ ^]^ making them very suitable for fundamental studies. These devices are generally fabricated with polydimethylsiloxane or thermoplastics through photolithography, and hydrogels can be introduced inside the chips for culturing cells in a 3D setting and under dynamic flow conditions.

Hydrogels can be engineered as microgel beads (generally with spherical shape), where cells can be encapsulated, forming a microtumor in each individual particle with a precise control of size, cell number and shape. These platforms allow the production of multicellular spheroids with tunable TME, including with cells that naturally do not form such spheroidal structures when cultured in 3D. Microgels also enable the immobilization of fully formed 3D spheroids in their ECM‐mimetic matrix, allowing to evaluate therapeutics immediately in fully formed MTs. These microtumors are compatible with HTS of anticancer therapeutics, as they enable the production of reproducible systems, where each hydrogel behaves as a small tumor surrogate.

The use of photopatterning in hydrogels allows the incorporation of microwells, where 3D spheroids can be maturated. In general, microwells are microengineered into a nonadhesive hydrogel (e.g., agarose). Cells can be deposited in these microwells forming cell aggregates that grow forming spheroids through cell–cell interactions.

### 3.2 Hydrogel Forming Biomaterials for Recreating the TME

Hydrogels for in vitro tumor modeling can be engineered from a wide range of natural biomaterials, such as polysaccharides (e.g., alginate, hyaluronic acid (HA), etc.), proteins (e.g., collagen, gelatin, fibronectin, laminin, fibroin, etc.), from synthetic materials including (PEG, self‐assembled peptides, etc.), or tissues/organs derived decellularized ECM (dECM, e.g., Matrigel, Geltrex, Cultrex, etc.). dECM hydrogels retain the biochemical complexity and signaling cues of the ECM, being composed by a complex mixture of proteins, especially collagens, glycosaminoglycans, proteoglycans, and GF, depending on the tissue/organ source.^[^ ^116^ ^]^

Matrigel has to date been the “gold standard” hydrogel for culturing cells in 3D and for in vitro tumor modeling,^[^ ^117^ ^]^ due to its spontaneous crosslinking at physiological temperature and to its biomolecular composition. This hydrogel precursor formulation is obtained from Engelbreth‐Holm‐Swarm mouse sarcoma tumors and it is generally comprised by collagen IV, laminin, and entactin, as well as proteoglycans and GFs.^[^ ^118^ ^]^ To reduce the impact of the variability in GFs content, a low GF formulation is also generally available commercially. However, the high batch‐to batch variability and the presence of several GFs that might mask the generated data are still key bottlenecks of such hydrogel platforms for in vitro disease modeling. Other decellularized tissues including the skin or breast, among others, have been also used for the fabrication of similar hydrogels.^[^ ^116^, ^119^ ^]^ Nonetheless, dECM‐based hydrogels are still relatively underexplored for developing in vitro tumor models.^[^ ^120^, ^121^, ^122^ ^]^

Protein hydrogels and dECM‐based hydrogels incorporate specific motifs for cells adhesion and enzymatic‐mediated matrix degradation which are essential to provide an adequate environment for cellular proliferation.^[^ ^123^ ^]^ They can also form random/aligned fibrous structures within the hydrogels, similar to collagen microfibers present in the ECM. Howbeit, in general, hydrogels mechanical properties do not recapitulate those of the native tumors, requiring further chemical modification and crosslinking, or combination with other reinforcing materials/structures.^[^ ^65^, ^124^, ^125^, ^126^ ^]^ Polysaccharide hydrogels are also extensively used for 3D cell culture due to their biocompatibility and biodegradability.^[^ ^127^ ^]^ Moreover, they are chemically versatile exhibiting a high amount of free hydroxyl, amine, or carboxylate groups, which are easily functionalized with other chemical moieties to improve hydrogels biochemical and mechanical properties. Interestingly, they can be inert (e.g., alginate) or have inherent biological activity (e.g., HA, chondroitin sulfate),^[^ ^127^, ^128^ ^]^ but they often require the functionalization with cell adhesion motifs to support cell adhesion/proliferation.^[^ ^65^ ^]^

On the other side, synthetic hydrogels comprising polymers like PEG derivatives, polyvinyl alcohol (PVA) or polymers of vinylated monomers enable a better control over scaffolds mechanical and chemical properties.^[^ ^82^ ^]^ They are considered inert, biocompatible materials, that do not significantly affect cellular viability/proliferation.^[^ ^82^ ^]^ Synthetic hydrogels do not exhibit cell degradation motifs, nor cell adhesion sequences, requiring further functionalization with bio‐functional moieties (e.g., peptides—cell adhesion and matrix metalloproteinase (MMP) degradable peptides) to imprint bioactive properties to such cell culture platforms.^[^ ^65^ ^]^ Under specific conditions, some synthetic polypeptides can self‐assemble to form hydrogels suitable for 3D cell culture.^[^ ^129^ ^]^ Similarly to synthetic polymers, they are easy to modify allowing the inclusion of functional residues that alter their bioactivity, biodegradability and physical properties.^[^ ^129^ ^]^ The synthetic origin of engineered peptides and polymers reduces batch‐to‐batch variability in comparison to that obtained with nature‐derived biomaterials.

Protein hydrogels and dECM can also be combined with synthetic polymers or polysaccharides, to form hybrid hydrogels. This can be materialized by simple blending (e.g., interpenetrating networks) or via covalent crosslinking. Hybrid hydrogels combine the physical properties and inert behavior of synthetic hydrogels and the biological features of proteinaceous hydrogels, such as cell‐mediated biodegradability or cell adhesion motifs. This enables a fine tuning of hydrogels mechanical properties or enables the addition of cell adhesion motifs presentation to other materials that generally lack such sequences.

## 4 Engineering the TME Using Protein‐Based Hydrogels

Some proteins have the ability to gel under specific conditions, forming highly biocompatible materials where cells can adhere and proliferate in 3D. Collagen, gelatin, fibrin, and silk‐derived proteins are widely used biomaterials to formulate hydrogels for 3D in vitro disease modeling. In the following sections seminal studies employing such materials are presented and discussed in light of their contribution for establishing ever more physiomimetic tumor models.

### 4.1 Collagen Hydrogels

Collagen is a natural protein and one of the major components of the ECM, with structural, biochemical, and cellular instructive functions in human tissues.^[^ ^130^ ^]^ There are 29 different types of collagen that have been identified to date, with collagen I (Col I) being the most abundant in human tissues and organs, and especially important in the TME of various solid tumors.^[^ ^130^ ^]^ Collagen is one of the main components of the TME and its production is regulated by both cancer and stromal cells.^[^ ^131^ ^]^ Modifications to collagen content and crosslinking/structure in the ECM (e.g., via MMP‐mediated degradation, enzyme‐mediated crosslinking or de novo deposition) may contribute to tumor progression and cancer MDR.^[^ ^131^ ^]^ Taking this into consideration, researchers have been focusing on better understanding the role played by collagens in cancer, and the way it can be recapitulated and manipulated toward assisting in the development more realistic 3D in vitro models for screening new therapeutics.^[^ ^131^ ^]^

#### 4.1.1 Collagen I Hydrogels

Col I self‐assembles into fiber‐forming biocompatible hydrogels.^[^ ^130^ ^]^ Owing to this feature, collagen‐based platforms have been extensively used as a substrate for cell adhesion/proliferation for numerous biomedical applications including tissue engineering and in vitro disease modeling. Col I formulation versatility is evidence by the diverse types of platforms based on collagen such as nanofibers, microspheres, hydrogels, or sponges^[^ ^132^ ^]^ and used in the field of wound healing,^[^ ^133^ ^]^ bone,^[^ ^134^, ^135^ ^]^ cartilage^[^ ^136^ ^]^ or neural^[^ ^137^ ^]^ regeneration, among others.^[^ ^132^ ^]^ This biomaterial can be obtained from several sources (e.g., rats, large mammals, fishes, avians, etc.),^[^ ^130^ ^]^ being most commonly obtained from the tendon of rat tails.

Col I inherently contains key cell adhesive domains (i.e., Arg‐glycine (Gly)‐Asp, RGD), that can be degraded via cell‐mediated degradation via MMP enzymes action. Col I hydrogels can be obtained by neutralizing the pH of the acid collagen solution and incubating at 37 °C, a crosslinking mechanism that is highly compatible with cell encapsulation. Col I can be used as a standalone 3D hydrogel for tumor models establishment, or be combined with other biomaterials to generate multifunctional hydrogels with improved ECM‐mimetic properties that can be valuable for in vitro modeling of a number of cancers including breast cancer,^[^ ^44^, ^138^, ^139^, ^140^, ^141^, ^142^, ^143^, ^144^ ^]^ colorectal cancer,^[^ ^145^, ^146^ ^]^ glioblastoma multiform (GBM),^[^ ^124^, ^147^, ^148^ ^]^ liver,^[^ ^149^, ^150^, ^151^ ^]^ lung cancer,^[^ ^111^, ^152^, ^153^ ^]^ osteosarcoma^[^ ^154^, ^155^ ^]^ or ovarian cancer,^[^ ^156^ ^]^ among others. Col I hydrogel stiffness can be easily manipulated by tuning the hydrogel precursor solution concentration, covalent crosslinking density and the chemical moieties involved in this crosslink. By exploring covalent crosslinks, promoting collagen fibers alignment, or by increasing its concentration and the crosslinking density, stiffer hydrogels can be obtained.^[^ ^157^, ^158^ ^]^ **Table** **1** summarizes relevant studies describing the use of collagen for in vitro tumor modeling.

**Table 1:** Collagen and collagen‐biomaterial hydrogel hybrids used for in vitro tumor modeling. Abbreviations: cancer‐associated fibroblasts (CAFs), collagen I (Col I), epithelial‐mesenchymal transition (EMT), extracellular matrix (ECM), human adipose‐derived mesenchymal/stromal stem cell (hAMSC), human bone marrow‐derived mesenchymal/stromal stem cells (hBM‐MSC), human mammary fibroblasts (HMFs), hyaluronic acid (HA), hypoxia induced factors 1*α* (HIF‐1*α*), matrix metallopeptidase (MMP), microtissues (MTs), nanoparticles (NPs), natural killers (NKs), polyethylene glycol (PEG), poly(ethyleneglycol)‐di(succinic acid *N*‐hydroxysuccinimide ester (PEG‐diNHS), telomerase‐immortalized human microvascular endothelial cell line (TIME), tumor‐on‐a‐chip models (TOC), and vascular endothelial growth factor (VEGF)

3D In vitro tumor model	Hydrogel composition	Model cells	Application	Achievements	Ref.
Breast cancer	Col I	MDA‐MB‐231	Hypoxia	Hydrogels of Col I at 8 mg mL^−1^ limit the O_2_ diffusion across the hydrogel, upregulating HIF‐1a and VEGF.	^[^ ^44^ ^]^
	Col I	MDA‐MB‐231/MCF‐7/TIME	Angiogenesis	Hydrogel to study cancer cell promoted angiogenesis. The system has three layers: a layer encapsulating cancer cells, an acellular layer and endothelial cells (TIME) layer on top. TIME cells have a more invasive phenotype when cultured with metastatic cells (MDA‐MB‐231) than nonmetastatic (MCF‐7).	^[^ ^138^ ^]^
	Col I	Premalignant mammary epithelial cell organoids	Invasion and migration	Hydrogels with stiffness between 0.4 and 4 kPa were obtained by varying collagen concentration from 1–7 mg mL^−1^ without affecting the porosity, using a 3D tension bioreactor system. Increased stiffness, increased cells invasion behavior.	^[^ ^139^ ^]^
	Col I	MDA‐MB‐231	Mechanobiology: interstitial flow fluid stresses	Microfluidic device to study the effect of interstitial flow fluid stress in cancer cells. These forces promoted a transcellular gradient in focal adhesion proteins and rheotaxis.	^[^ ^140^ ^]^
	Col I	MDA‐MB‐231	Mechanobiology: ECM biophysical properties	The outcome of anti‐migration drugs (Y27632, cytochalasin D, GM6001, nocodazole) depends on the matrix mechanics and architecture, showing the importance of studying ECM biophysical properties for screening dugs targeted to these mechanisms.	^[^ ^141^ ^]^
	Col I	MCF‐10A	Interstitial fluid pressure effects	Breast tumor 3D spheroids growth promoted a compressive remodeling of collagen hydrogel through fiber bending and the rupture of crosslinks, related with a higher interstitial fluid pressure.	^[^ ^162^ ^]^
	Col I	HUVEC/MDA‐MB‐231	Interaction between endothelial and cancer cells	A microfluidic device recreating microvasculature was fabricated by a pneumatic microchannel network method and filled with a Col I hydrogel. Breast cancer cells showed adhesive interactions with the endothelial cells.	^[^ ^142^ ^]^
	Col I	MDA‐MB‐231/TIME	Effect of fluid forces	Microfluidic tumor vascular model to study the effect of different fluid flow on the interactions between tumor/endothelial cells.	^[^ ^143^ ^]^
	Col I	MDA‐MB‐231/MCF‐7	Effect of ECM alignment in cancer cells behavior.	Microchip where hydrogels were aspirated/ejected to produce dense and aligned collagen fibers. MDA‐MB‐231 cells aligned in the direction of the fibers, whereas MCF‐7 cells remained as spheroids aligned to the fiber direction.	^[^ ^144^ ^]^
	Col I	HT‐29/HCT‐116/DLD‐1/AsPC‐1/Panc‐1	3D Spheroids assembly	Mini‐pillar array chip for generating 3D tumor spheroids in alginate, collagen, or Matrigel hydrogels. Compatible with in situ histological analysis.	^[^ ^183^ ^]^
	Col I	CAFs/MDA‐MB‐231	Drug screening	Microwell array containing a hydrogel laden with CAFs and cancer cells. A combination of tranilast and doxorubicin reduced 3D spheroids size, as well as reduced ECM stiffness.	^[^ ^167^ ^]^
	Col I + alginate	MCF‐7/HUVEC/hAMSC	Vascularized tumor MTs	Microcapsules with a collagen core encapsulating cancer cells and a shell of alginate were used as a building unit of a 3D MT. Microcapsules were assembled in presence of endothelial cells and hADSCs, creating vascularized tumor MTs. The MTs exhibited a high resistance to doxorubicin and NPs encapsulating doxorubicin.	^[^ ^166^ ^]^
	Col I + alginate	HMFs/MDA‐MB‐231	Invasion and migration	Multicellular 3D spheroids were embedded in the hydrogel (4 kPa) to study cell migration of both cells (not observed in gels only made of collagen).	^[^ ^174^ ^]^
	Col I + alginate	CAFs MDA‐MB‐231	Mechanobiology: mechanoregulation	Compliant hydrogels (108 Pa) enabled cell spreading and activated its protumorigenic paracrine activity, through YAP/TAZ protein.	^[^ ^175^ ^]^
	Col I + alginate	MCF‐7/MDA‐MB‐231/Adipocytes	Effect of adipocytes in tumor invasion	Microbeads of 2.7 kPa stiffness were obtained with laser direct‐write technology. Breast cancer cells were encapsulated in microbeads and then embedded in a hydrogel containing adipocytes. Cancer cells invasiveness was higher when using differentiated adipocytes from obese patients in comparison to those of nonobese patients.	^[^ ^184^ ^]^
	Col I + alginate + agarose	MCF‐7	Cell proliferation	Hydrogels of 1 kPa cultured under rotary cell culture enabled the formation of 3D spheroids with high proliferation promoted by the activation of the ERK1/2‐MAPK pathway.	^[^ ^185^ ^]^
	Col I + alginate + oligomeric silsesquioxanes	HMF/MDA‐MB‐231	Invasion and migration	HMFs mamospheres were cultured in hydrogels of 2.8–4.4 kPa containing MDA‐MB‐231 cells. Fibroblasts were able to remodel collagen microstructure and facilitated cancer cells invasion.	^[^ ^176^ ^]^
	Col I + agarose	HCT116/PPT2/MCF7/LNCaP/A172	3D spheroids formation	Array with low adhesion microchambers to generate 3D spheroids, enabling their real‐time observation.	^[^ ^186^ ^]^
Breast‐to‐bone metastasis	Col I	MDA‐MB‐231/MC3T3‐E1 (preosteoblasts)	Stromal/cancer cell interactions	Cancer cells or its conditioned media impaired osteoblasts differentiation and reduced mineralization.	^[^ ^169^ ^]^
	Col I	hBM‐MSCs osteo differentiated/HUVEC/MDA‐MB‐231	Specificity of human breast cancer metastases to bone	TOC of vascularized bone microenvironment to study the tumor trans‐endothelial migration toward bone tissues. Cancer cells extravasated to the bone microenvironment.	^[^ ^115^ ^]^
Cervical cancer	Col I	HeLa/NK‐92	3D cytotoxicity assay for NK	Hydrogels encapsulating cancer cells were included in the microdevice and perfused with NK cells. This platform showed that the migration of NKs inside the gel was reduced, reducing their cytotoxic activity in comparison to that obtained with 2D monolayer models.	^[^ ^172^ ^]^
Colorectal cancer	Col I	HT‐29/CCD‐18Co	Cross‐talk CAFs/cancer cells	Microfluidic device where 3D tumor spheroids are in close proximity to CAFs. 3D spheroids exhibited a larger size and displayed a higher fibronectin production, whereas CAFs showed an increase in the migration behavior. 3D spheroids were also more resistant to paclitaxel in the presence of CAFs.	^[^ ^110^ ^]^
	Col I	HCT‐116	Nutrients gradients	TOC where nutrient and pH gradients were recreated without any oxygen deprivation, cancer cells upregulated genes related with stress and survival.	^[^ ^171^ ^]^
	Col I + alginate	CT26	Spheroid formation for HTS	Fabrication of hydrogel microcapsules by a compound jet‐in‐air, with a liquid core and compatible with HTS.	^[^ ^126^ ^]^
	Col I + Matrigel	SW480	Effect of shear modulus	A laser speckle contrast shear wave elastography was used to evaluate the shear modulus of the hydrogels, and a correlation with the fiber density was obtained. Researchers could track spatiotemporal modifications of hydrogels stiffness.	^[^ ^187^ ^]^
Glioblastoma	Col I	U‐251 MG	Model of blood vessel obstruction	Microdevice that recreates the blood vessel obstruction that generally occurs in GBM. It was observed that after 6 days, GBM cells migrated toward the perfused channel forming a pseudopalisade‐front.	^[^ ^145^ ^]^
	Col I	U251‐MG	Epigenomic analysis	The culture in 3D and oxygen has an impact in histone modification patterns, with the phenotype obtained in 3D being more similar to the in vivo setting, than in 2D.	^[^ ^146^ ^]^
	Col I + agarose	HUVEC/UC‐MSC/SH‐SY5Y	Vascularize tumor	Use of a drop‐on‐demand and layer‐by‐layer bioprinting approach to 3D bioprint the vasculature, stroma and cancer rosettes components of GBM in a hydrogel.	^[^ ^147^ ^]^
	Col + HA	Patient tumor derived OSU‐2 cell culture	Invasion and migration	The type of collagen and HA concentration used in the hydrogel had an impact in the morphology of cancer cells. In Col IV, cells exhibit a round morphology, meanwhile in Col I or III they have a spindle‐shape. Higher HA concentrations promoted t cell migration.	^[^ ^148^ ^]^
	Col I crosslinked with 8‐arm PEG Succinimidyl Glutarate (hexaglycerol)	U87 A172	Invasion and migration	3D tumor spheroids exhibited a higher migration speed and overexpression of MMP‐2, MMP‐9, urokinase plasminogen activator and tissue plasminogen activator when cells were cultured in non‐crosslinked hydrogels. Aprotinin and tranexamic acid reduced 3D spheroids migration, but not in 2D cultures.	^[^ ^124^ ^]^
	Col I functionalized with GRGDSPC (specific for integrin avb3)	RAW264.7 C166 GL261	Role of macrophages in GBM angiogenesis	GBM angiogenesis microchip model, integrating macrophages, endothelial cells and an ECM‐mimetic hydrogel. The constructs polarized macrophages toward an M2‐like phenotype, which promoted angiogenesis and immunosuppression. A key role of TGF‐*β*1 and surface integrin (*α*5*β*3) was required for establishing this immunosuppressive environment.	^[^ ^188^ ^]^
	Col I + HA	KIM‐2 adipocytes	Organoid formation	Cells can recreate a physiologically relevant mammary gland, showing polarized ductal and acinar structures, being the collagen/HA ratio fundamental in the organoid formation. Higher HA concentration enables the acinar development.	^[^ ^178^ ^]^
Glioblastoma and ostosarcoma	Col I‐MA + HA‐SH	Patient‐derived organoids of GBM and osteosarcoma	Drug screening	96‐well plate platform compatible with HTS of anticancer drugs. Through a 3D printing approach, spheroid hydrogel organoids were formed within each well. The HTS in vitro drug testing proved the applicability of this model for personalized treatment of GBM.	^[^ ^179^ ^]^
Hepatic cancer	Col I	HepG2/3T3‐J2	Drug screening	Spheroids of fibroblasts and liver carcinoma cells were encapsulated in the hydrogel, and showed a higher resistance to anticancer drugs than 2D cultures and spheroids of cancer cells.	^[^ ^149^ ^]^
	Col I + PEG‐diNHS	HepG2	Effect of the stiffness	Differences in the hydrogel stiffness could alter the phenotype of cancer liver cells from malignant 3D spheroids (0.7 kPa) to 3D hepatoids where their malignancy was suppressed (4 kPa).	^[^ ^150^ ^]^
	Col I + PEG‐diNHS	HepG2	Effect of stiffness	Effect of MMP‐1 in hepatocarcinoma 3D tumor spheroids. MMP degradation modified the elastic modulus from 4 to 0.5 kPa, and promoted cancer cells proliferation, while at the same time reduced the expression of E‐cadherin and detoxification capacity.	^[^ ^151^ ^]^
Intrahepatic cholangiocarcinoma	Col I	Cancer‐associated myofibroblasts cholangiocarci‐noma cell	Desmoplasia	Model of desmoplasia that promoted cell proliferation, formation of a dense fibrocollagenous stroma, cholangiocarcinoma cell anaplasia.	^[^ ^189^ ^]^
Lung cancer	Col I	H1299	Effect of the ECM and TGF‐*β* in tumor progression	Microfluidic device to study cell invasiveness. Higher collagen concentrations promoted the formation of spheroids and TGF‐*β* can induce spheroid‐like or strand‐like morphology depending on its concentration. Higher TGF‐*β* concentrations increase the invasiveness capacities.	^[^ ^111^ ^]^
	Col I	A549	Drug screening	Lung tumor 3D spheroids with a tissue‐like morphology, an increased EGF/EGFR expression and reduced sensitivity to anticancer drugs.	^[^ ^152^ ^]^
	Col I + HA	Pleural effusion aspirate of lung adenocarcinoma	Drug screening	Hydrogels support lung adenocarcinoma organoids growth with a lower sensitivity to chemotherapeutic drugs than in 2D.	^[^ ^153^ ^]^
	Col I + Matrigel	H1299	Effect of the stiffness	Increase in stiffness (achieved by higher Matrigel concentration) from 44 to 513 Pa promoted the expression of *β*1 integrin and increased MMP activity. The ECM remodeling induced fibers alignment and compaction, which in turn increased tractions in cells, but did not increased cells motility.	^[^ ^190^ ^]^
Osteosarcoma	Col I	hFOB1.19 MG63	Effect of stiffness and adherent molecules in the osteogenesis and tumorigenesis	Osteosarcoma cells cultured in Col I (3.4 MPa) and agarose (30 MPa) exhibited a more tumorigenic phenotype than in Matrigel (0.7 MPa) or alginate (0.6 MPa), indicating that variations in elasticity have an impact in tumorigenesis rather than cell adhesion. Osteoblast showed a better osteogenic differentiation in Matrigel and Col I, showing that cells adhesion to the ECM is more important than hydrogels stiffness.	^[^ ^154^ ^]^
Ovarian cancer	Col I	OvCa429/OvCa433/DOV13	Effect of compression forces	Long‐term compression forces upregulated the expression of EMT genes related in the dissemination of epithelial ovarian cancer in Multicellular spheroids. Improved the lateral dispersal of OvCa433 cells.	^[^ ^156^ ^]^
Sarcoma	Col I	Mouse undifferentiated pleomorphic sarcoma cells	Invasion and migration	Hypoxic gradients and different Col I fiber densities were recapitulated in hydrogels. In hypoxic condition, higher fiber densities had a positive effect in tumor migration and matrix degradation. In nonhypoxic conditions, this effect was not observed.	^[^ ^155^ ^]^
Skin adenocarcinoma	Collagen 1 + PEG‐diNHS	Human fibroblasts CCD‐1065Sk	Effect of stiffness	Hydrogel stiffness is sufficient to activate fibroblast through the production of soluble factors. C‐C chemokine receptor type 4 and b1 and b3 integrins were used to transduce these signals. Researchers observed that focal adhesion kinase, phosphoinositide 3‐kinase and palladin were involved in the activation pathways.	^[^ ^180^ ^]^

**Figure 3:**
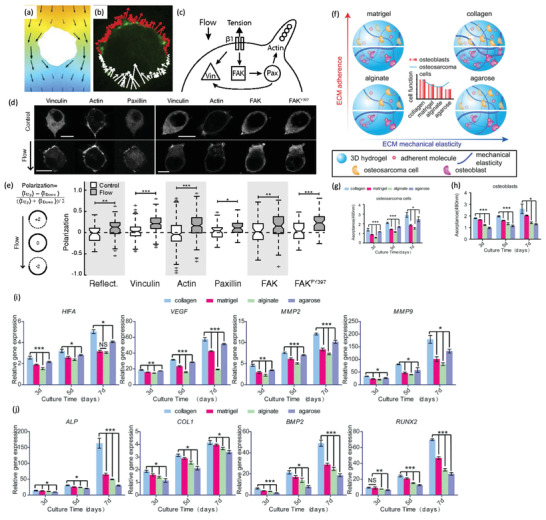
Collagen hydrogels used for cancer mechanotransduction and in vitro cancer modeling. a–e) Effect of interstitial flow fluid stress in MDA‐MB‐231 cancer cells laden in a collagen I hydrogel. a) Simulation of flow (velocity 4.6 µm s^−1^) across the cell. Pressure heat map. Orange coding represents the maximum and blue the minimum, and black arrows the local fluid velocity vectors. b) Micrograph of the reaction forces. Red arrows represent the tensile forces. White arrows represent compressive forces. Green channel represents vinculin distribution in collagen hydrogel laden cells subjected to interstitial flow fluid stress. These forces are required to maintain the static condition. A clear vinculin positive staining is observed in the area of cell/hydrogel tension. c) Scheme showing that integrin activation by interstitial flow fluid stress induces the localization of vinculin, FAK, paxillin, and actin at the upstream, and the formation of a protrusion in this direction. d) Vinculin, actin, paxillin, FAK, and FAKPY397 localization in a cell subjected, and not subjected to interstitial flow fluid stress. The flow promotes the polarization of the proteins upstream (scale bar: 10 µm). e) Evaluation of vinculin, paxillin, FAK, and FAKPY397 polarization at 4.6 µm s^−1^ for 4 h relative to upstream fluorescence intensity (+2 represents the maximum upstream polarization, and −2 represents the maximum downstream polarization). (^***^ *p* < 0.001, ^**^ *p* < 0.01, and *p < 0.05). Reproduced with permission.^[^ ^140^ ^]^ Copyright 2014, National Academy of Sciences. f–j) ECM influence in osteoblast and osteosarcoma cells. f) Scheme of the tested platforms. g) Osteosarcoma and h) osteoblast cell proliferation Col I, agarose, Matrigel, and alginate hydrogels (*p < 0.05, ^***^ *p* < 0.001). i) mRNA expression of HIFA, VEGF, MMP2, and MMP9 of osteosarcoma MG‐63. j) mRNA expression of ALP, COL1, BMP2, and RUNX2 of osteoblast hFOB1.19 (*p < 0.05, ^**^ *p* < 0.01, ^***^ *p* < 0.001). Reproduced with permission.^[^ ^154^ ^]^ Copyright 2019, Wiley‐VCH GmbH & Co.

In fundamental cancer research, Col I has been widely used to study mechanobiology and the effect of stiffness and porosity, in the tumorigenesis and invasion behavior of malignant cells.^[^ ^111^, ^140^, ^141^, ^154^, ^159^ ^]^ It is well known that cancer cells can sense their ECM‐mimetic subtract stiffness, changing their phenotype and proliferation as a response.^[^ ^160^ ^]^ Compression forces induced by interstitial fluid pressurization or by tumor growth have shown their importance in tumor progression due to mechanotransduction.^[^ ^161^ ^]^ To better understand this phenomenon, researchers have studied the effect of high intraperitoneal pressure, a hallmark of ovarian cancer, in the dissemination of epithelial ovarian cancer cells. Cancer cells dissemination generally occurs by the invasion of the peritoneal cavity during which cells proliferate and cluster into multicellular aggregates that secrete ascites fluid, leading to a bad prognosis.^[^ ^156^ ^]^ 3D multicellular aggregates of OvCa429, OvCa433, and DOV13 cells were initially developed and then incorporated into collagen hydrogels to test the effect of compression forces. It was observed that long‐term exposure to compression, upregulated the expression of genes related with epithelial‐mesenchymal transition and improved the lateral dispersal of OvCa433 cells.^[^ ^156^ ^]^ Col I fibers alignment and accumulation are also well described to have a tremendous impact in tumor progression and metastasis. In this context, it was shown that breast 3D tumor spheroids growth promoted a compressive remodeling of the collagen hydrogel network where they were established. This phenomenon was caused by fiber bending and the rupture of collagen crosslinks, related with a higher interstitial fluid pressure exerted by the growing microtumors.^[^ ^162^ ^]^ Another interesting study also demonstrated that collagen hydrogels can be used to study the effect of interstitial flow fluid stresses into cancer cells. In solid tumors, there is an interstitial fluid pressure that provokes fluid movement from the nuclei of the tumor, and it has a rheotaxis effect on cancer cells (**Figure** **3**a–e). By using a microfluidic device and metastatic breast cancer MDA‐MB 231 cells, researchers were shown that interstitial flow fluid stress promoted the establishment of a transcellular gradient in focal adhesion proteins (vinculin, paxillin, FAK, FAKPY397, and *α*‐actinin), and the formation of protrusions in the direction of the upstream cell side, and rheotaxis.^[^ ^140^ ^]^

Col I hydrogels have also been widely used to bioengineer different types of 3D in vitro solid tumor models of highly prevalent malignancies such as breast cancer. For example, the culture of the metastatic breast cancer cell line MDA‐MB‐231 in Col I hydrogels (8 mg mL^−1^) was recently used as an approach to support cellular proliferation and the establishment of a biomimetic tumor model.^[^ ^44^ ^]^ Cells were cultured under static conditions in contrast with the dynamic culture followed when using microfluidic devices. Recent studies have suggested that cells cultured under dynamic conditions show a higher cellular proliferation but still show the same morphology and behavior as cells cultured under static conditions.^[^ ^163^ ^]^ Importantly, by using this approach, researchers were able to observe a limitation in oxygen and nutrients diffusion across the hydrogel, and at ≈150–200 µm depth an upregulation in the expression of HIF‐1*α* was obtained. This was accompanied by an upregulation of vascular endothelial growth factor (VEGF)‐A expression, recreating the VEGF‐A expression promoted by HIF‐1*α* similarly to that occurring in vivo,^[^ ^164^ ^]^ suggesting the angiogenic potential of the tumor model, since VEGF‐A is involved in tumor angiogenesis.^[^ ^44^ ^]^ This evidences that collagen hydrogels are suitable to recreate the hypoxic environment that appears in solid tumors at a depth of ≈150 µm,^[^ ^165^ ^]^ being the limitation of oxygen and nutrients diffusion imposed by the matrix as it occurs in vivo and not by a control of the airflow as performed in other hypoxia models.

**Figure 4:**
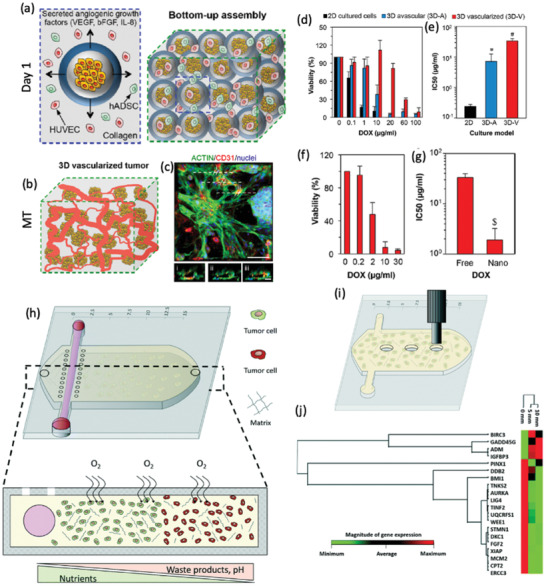
Microtumor and microfluidic device comprising Col I hydrogels for 3D in vitro cancer modeling. a–g) Use of Col I and alginate for the preparation of tumor MTs for drug screening. a,b) Schematic representation of the microcapsules used to create the tumor MT, microcapsules are formed by a shell of alginate and a nucleus of Col I encapsulating MCF‐7 cancer cells. The produced microcapsules are then self‐assembled in the presence of human umbilical vein endothelial cells and human adipose‐derived mesenchymal/stromal stem cells in Col I hydrogel in the microfluidic perfusion device. c) Vessel structure staining in the MTs (green channel: actin, red channel: CD31, and blue channel: cell nuclei) and the cross‐sectional images (i–iii) showing the vessels lumen (scale bar: 50 µm, for cross‐sections 20 µm). d) Viability and e) IC50 of cells cultured in 2D, 3D avascular, and 3D vascular MTs after 4 days of treatment with free doxorubicin. f) Cell viability of vascular MT after 4 days of treatment with NPs encapsulating doxorubicin. g) IC50 of free and nanoencapsulated doxorubicin in vascular MT after 4 days of treatment. Reproduced with permission.^[^ ^166^ ^]^ Copyright 2017, American Chemical Society. h–j) Simulation of oxygen and nutrients gradients in a colorectal microfluidic model. h) Scheme of the microdevice containing HCT‐116 cell‐laden collagen I hydrogels. The cell density seeded in the hydrogel modulates the necrotic region (green: life cells, red: death cells). i,j) Gene expression changes at different hydrogel locations. i) Hydrogel punches were isolated from different zones of the hydrogel for studying the gene profile. j) Gene expression profile indicating that cells express different genes in the first 5 mm of the hydrogel, than those located at 10 and 15 mm. Reproduced with permission.^[^ ^171^ ^]^ Copyright 2001, Royal Society of Chemistry.

In a different approach, modular tumor MTs were generated by using Col I microcapsules as building units (**Figure** **4**a–g).^[^ ^166^ ^]^ The microcapsules comprised an alginate shell and a collagen core encapsulating breast cancer cells (MCF‐7). The microcapsules were assembled in the presence of endothelial cells (human umbilical vein cells, HUVEC) and human adipose mesenchymal/stromal stem cells (hAMSCs), recreating vascularized microtumor tissues. This platform was explored for testing the anti‐tumor performance of NPs containing doxorubicin and free doxorubicin. Interestingly, it was observed that MTs were 13.2 and 4.2‐fold more resistant to drug‐loaded NPs and free drug administration respectively,^[^ ^166^ ^]^ further demonstrating the importance of the stroma in the treatment response. Collagen has also been used as a hydrogel for breast cancer modeling in well arrays. Col I hydrogels laden with cancer‐associated fibroblasts (CAFs) were microengineered to introduce wells into the structure, by using a stamp. Afterward, MDA‐MB‐231 metastatic breast cancer cells were seeded in the stamped wells. This platform was validated by assessing the performance of two anticancer drugs (tranilast and doxorubicin) in the context of tumor fibrosis. By using this elegant set up, it was observed that the combination of both anticancer drugs elicited a reduction of tumor growth and invasion, as well as a reduction in the overall stiffness induced by a decrease in the collagen density and fibronectin disruption.^[^ ^167^ ^]^ These findings further evidence the importance of recapitulating the tumor stroma to learn about the action mechanisms of new therapeutics, as the results outcome is influenced by the proper recapitulation of the TME.

Collagen hydrogels have also been extensively used as ECM‐mimetic platforms to establish microtumor models and evaluate cancer cells invasiveness in vitro, in 3D. Using these hydrogels, researchers showed the impact of ECM stiffness in cancer cells invasion in a mode independent of ECM porosity or architecture.^[^ ^139^ ^]^ Such was accomplished by using a 3D tension bioreactor to fabricate Col I hydrogels at different collagen concentrations (1–7 mg mL^−1^), with different stiffness (0.4–4 kPa), while assuring a constant porosity. It has been reported that hydrogels with a Col I concentration up to 5 mg mL^−1^ lead to an increased cell invasiveness.^[^ ^168^ ^]^ However, above this concentration, invasiveness is limited owing to a reduction in hydrogel porosity. By using this platform, it was clear that when porosity is not a limiting factor, an increase in collagen stiffness promotes cell invasion of premalignant mammary epithelial cells.^[^ ^139^ ^]^

The effect of the collagen fiber density and hypoxic gradients in cellular migration of sarcoma cells was also evaluated in a different study where the oxygen diffusion across the matrix was controlled by tuning hydrogel's thickness.^[^ ^155^ ^]^ Through this strategy, it was observed that in nonhypoxic conditions, fibers density had no effect on the migration of mouse undifferentiated pleomorphic sarcoma cells. Howbeit, in hypoxic conditions, sarcoma cells migrated faster and degraded the matrix more rapidly in high fiber density scaffolds, compared to those exhibiting lower fiber density.^[^ ^155^ ^]^ It becomes clear that hydrogel architecture (i.e., collagen density and hydrogel size) does play a role in cell migration, an important parameter that must be taken into consideration when designing Col I‐based 3D in vitro tumor models for evaluating these key biological processes and pathways.

Col I hydrogels have also been recently explored for engineering biomimetic in vitro bone tissues for evaluating breast‐to‐bone metastasis^[^ ^169^ ^]^ or osteosarcoma.^[^ ^154^, ^170^ ^]^ Hydrogel platforms were used to compare the effect of the stiffness and adherent molecules in osteosarcoma osteogenesis and tumorigenesis, using four types of hydrogels Col I (3.4 MPa), Matrigel (0.7 MPa), agarose (3.0 MPa), and alginate (0.6 MPa) (Figure 3f–j). All hydrogels supported osteoblasts (hFOB1.19) and osteosarcoma cells (MG‐63) proliferation. However, osteosarcoma cells proliferated faster and exhibited a more tumorigenic phenotype in Col I and agarose hydrogels, being the variations in elasticity more relevant than cell adhesion for these cells. Osteoblasts showed a higher osteogenic differentiation in Matrigel and collagen, indicating that cells‐ECM adhesion cues are crucial for their functionality and bioactivity.^[^ ^154^ ^]^

Col I has also been used for recreating the tumor vasculature in microfluidic devices. Recently, researchers have fabricated a microchip where the cell culture medium flow through lateral microchannels could be controlled and blocked to recreate a blood‐vessel obstruction. This device recapitulated the blood vessel obstruction which occurs in GBM tumors. To capture this hallmark, researchers cultured GBM cells, U‐251 MG, in the Col I hydrogel and blocked one lateral channel, mimicking a vessel obstruction in one side of the chip. After 6 days of flow blocking, it was observed that GBM cells migrated toward the perfused channel forming a pseudopalisade‐front triggered by the lack of oxygen and nutrients.^[^ ^145^ ^]^ These researchers also fabricated a TOC where a nutrient gradient without any oxygen deprivation was recreated by introducing a blood vessel mimicking channel. Colorectal cancer cells were cultured in a Col I hydrogel inside the microdevice, and nutrients and pH gradients were available for promoting cellular proliferation (Figure 4h), which originated changes in the expression of genes related to stress and survival (Figure 4i–j).^[^ ^171^ ^]^ Park et al. also showed that microfluidic devices can be used to evaluate the cytotoxicity of natural killers (NK) against cancer cells in a 3D environment.^[^ ^172^ ^]^ In this approach, HeLa cells were initially embedded in Col I hydrogels and then NKs were introduced into the platform via the lateral channel. NKs migrated toward malignant cells, exerting a cytotoxic effect in the 3D collagen microenvironment. Indeed, it was demonstrated that 2D results differed from those obtained in 3D, and that stiff ECMs reduced NK migration in tumors, reducing their efficacy through a physical barrier effect without altering their cytotoxic activity.^[^ ^172^ ^]^

Apart from being suitable for in vitro modeling, collagen hydrogels can also be used for generating 3D tumors in vivo. In this context, ovarian cancer cells (SKOV3) laden in Col I hydrogels and injected in vivo, displayed a higher degree of similarity to human tumors when compared to those obtained with Matrigel, alginate or agarose hydrogels.^[^ ^173^ ^]^ Such Col I hydrogels originated tumors with the largest size and vascularization, as well as an increased MMP, HIF‐1*α* and VEGF‐A expression,^[^ ^173^ ^]^ evidencing the potential of this biomaterial to be used as an alternative to Matrigel for tumor implantation in rodent models.

#### 4.1.2 Hybrid Collagen hydrogels

**Figure 5:**
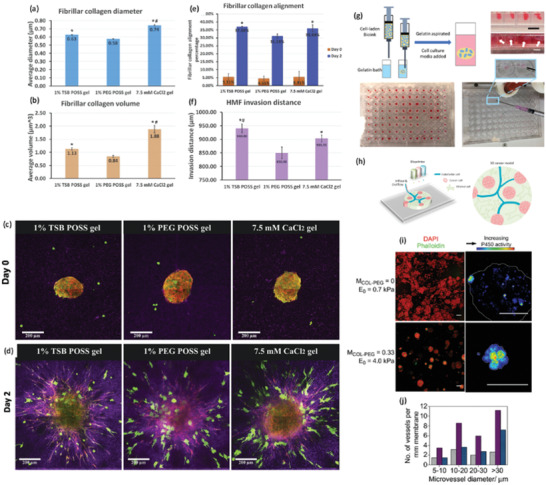
Hybrid collagen‐based hydrogels for 3D in vitro tumor modeling. a–f) Collagen remodeling by mamospheres comprising MDA‐MB‐231 and mammary fibroblasts in hydrogels of Col I/alginate containing different polyhedral oligomeric silsesquioxanes (POSS) (trisilanolisobutyl‐POSS (TSB POSS) or PEGylated‐POSS (PEG POSS). a,b) Collagen microstructure in 1% TSB POSS, 1% PEG POSS, or CaCl_2_ crosslinked (control) hydrogels. a) Collagen fiber diameter and b) volume, being *p > 0.05 in comparison with the PEG POSS and ^#^ *p* > 0.05 in comparison with the TSB POSS. c) Mamospheres embedded in hydrogels on day 0 showing no cell protrusions in the spheroids and d) after 2 days, where cells exhibited an elongated morphology and migration. 3D Spheroids are represented as a green/red overlay, individual MDA‐MB‐231 cells in green color, and a second harmonic imaging of fibrillar collagen in purple color. e,f) MDA‐MB‐231 invasion and fibers remodeling. Collagen fibers alignment perpendicular to spheroid on days e) 0 and 2 and f) invasion distances of cells on day 2 , being *p > 0.05 in comparison with the PEG POSS and ^#^ *p* > 0.05 in comparison with the control. Reproduced with permission.^[^ ^176^ ^]^ Copyright 2019, Elsevier. g) Fabrication of organoid spheroidal constructs made of collagen I methacrylate and thiolated‐HA by 3D‐printing technology in a gelatin bath compatible with HTS. Gelatin can be afterward removed to have the organoids. Reproduced with permission.^[^ ^179^ ^]^ Copyright 2020, by the authors. h) CAD design of a tumor model for 3D printing and its final result after printing. The hydrogel was produced by using agarose and collagen I and mixed with different food colorants. Reproduced under the terms of the Creative Commons Attribution license CC‐BY 4.0.^[^ ^147^ ^]^ Copyright 2019,The Authors, published by MDPI. i,j) Effect of the stiffness on HepG2 cells. Stiffness increase in the hydrogels was achieved by adding rising amounts of PEG‐diNHS (PEG‐COL). i) Morphology of the spheroids and effects of stiffness on detoxification activity. HepG2 spheroids formed in collagen hydrogels (*E*_0_ = 0.7 kPa, first row) and in collagen‐PEG gels (*E*_0_ = 4.0 kPa, second row). The first column depicts 3D spheroids morphology. Green channel represents phalloidin‐Alexa 488 labeled actin and red channel represents nuclei staining with DAPI. In the right column, 3D spheroids live imaging of the activity of P450. The fluorescent images were processed to have 256 pseudocolors representing its activity. Lower stiffness promoted the formation of larger, more disorganized spheroids than higher stiffness substrates. 3D spheroids cultured in collagen hydrogels of 0.7 kPa exhibited a lower cytochrome P450 detoxification activity and generated larger and more disorganized 3D spheroids than in collagen‐PEG hydrogel of 4 kPa (scale bar: 50 µm). j) Effects of hydrogel stiffness in proangiogenic activity. Size and number of blood vessels formed in chicken CAM after the implantation of hydrogel embedded HepG2 3D spheroids. Purple channel represents pure collagen hydrogels (0.7 kPa) laden with spheroids, blue channel represents collagen‐PEG hydrogels (4.0 kPa) laden with 3D spheroids and gray channel represents, acellular pure collagen hydrogels (0.7 kPa). Reproduced with permission.^[^ ^150^ ^]^ Copyright 2011, Elsevier.

Col I has also been combined with other biomaterials to improve its stability, stiffness, or bioactivity. Table 1 summarizes important studies addressing Col I and its combination with other biomaterials, as well as the main findings. One of the most widely used polymers combined with collagen is alginate, due to its facile and immediate crosslinking via divalent ions (e.g., Ca^2+^). This combination enables the formulation of 3D hydrogels with excellent biocompatibility and permeability, with tunable stiffness, being appropriate to evaluate cancer cell invasion^[^ ^174^ ^]^ or mechanobiology.^[^ ^175^ ^]^ In an elegant approach, nanosized polyhedral oligomeric silsesquioxanes were introduced in Col I/alginate hydrogels to study the effect of matrix remodeling in cell migration (**Figure** **5**a–f).^[^ ^176^ ^]^ The combination of these materials enabled the generation of different fibrillar collagen structures, while assuring constant stiffness and porosity. Human mammary fibroblasts (HMFs) and MDA‐MB‐231 mamospheres were cultured in such hydrogels, which stimulated fibroblasts to remodel the collagen microstructure, facilitating cancer cells invasion.^[^ ^176^ ^]^ Col I/alginate hydrogel platforms were also explored to evaluate ECM remodeling influence in the cross‐talk between CAFs and cancer cells. For this purpose, researchers synthetized hydrogels with stiffness ranging from 108 to 898 Pa, and recapitulated the breast TME using CAFs and metastatic breast cancer cells (MDA‐MB‐231). Interestingly, more compliant hydrogels, in contrast with rigid ones, enabled a spindle‐shaped cell morphology and activated protumorigenic paracrine activity, as demonstrated by nuclear translocation and higher expression of YAP/TAZ proto‐oncogene proteins (related with mechanosensing).^[^ ^175^ ^]^

Col I has also been combined with HA, particularly for generating GBM in vitro models, as this biopolymer is the main component of the brain ECM and is overexpressed in GBM cells.^[^ ^177^ ^]^ By exploring different types and concentrations of collagen and HA researchers were able to study the cell migration in these tumors.^[^ ^148^ ^]^ Remarkably, the type of collagen used for hydrogel platforms fabrication had an impact in patient‐derived cancer cells morphology. In Col IV, cells exhibited a round morphology, whereas in Col I, III hydrogels a spindle‐shaped morphology was observed. These researchers also discovered that higher HA concentrations promoted cell migration. All these results point out the importance of the material used for hydrogel formulation when studying GBM.^[^ ^148^ ^]^ On the other hand, Col I/HA hydrogels can also be used for recreating physiologically relevant mammary glands, showing polarized ductal and acinar structures^[^ ^178^ ^]^ In this context, it was recently shown that Col I/HA hydrogels were able to sustain the growth of lung adenocarcinoma 3D organoids, to maintain their phenotype and allowing them to display an anatomy similar to that found in vivo. The generated in vitro models exhibited lower sensitivity to chemotherapeutic agents than that obtained in standard 2D cell cultures, further corroborating the importance of the 3D set up and of the inclusion of ECM‐mimetic elements.^[^ ^153^ ^]^

Collagen has also been combined with agarose or HA to form bioinks in the context of 3D bioprinted in vitro tumor models fabrication.^[^ ^147^, ^179^ ^]^ In a recent report, researchers used a drop‐on‐demand and additive manufacturing approach to 3D bioprint components of the tumor vasculature (HUVEC), stroma (umbilical cord‐derived mesenchymal/stromal cells, UC‐MSC), as well as GBM cancer rosettes (human bone marrow‐derived epithelial‐neuroblastoma immortalized cells, SH‐SY5Y) as show in figure 5h. Although stromal cells and cancer cells proliferated within the hydrogel matrix, HUVECs were not able to recreate vasculature in the fabricated microtumor, most likely owing to the small size of the formed rosettes.^[^ ^147^ ^]^ Advanced 3D bioprinting was also explored to develop HTS platforms based on collagen/HA hydrogels. In this approach 3D spheroid hydrogel organoids were deposited into a supporting gelatin hydrogel confined in 96‐well plates, avoiding bioink spreading in the well. This HTS platform was used for patient‐derived organoids of GBM and osteosarcoma, and even though some variability was obtained depending on the drug and patient tested, in vitro models drug response was validated, proving its potential use as a screening platform (Figure 5g).^[^ ^179^ ^]^

In addition to naturally available biomaterials, also synthetic polymers have been used in combination with Col I, particularly PEG, specifically PEG‐di(succinic acid *N*‐hydroxysuccinimide ester) (PEG‐diNHS) that can be used to covalently crosslink the amine groups present in collagen chains.^[^ ^151^, ^180^ ^]^ This platform is interesting to test the effect of collagen stiffness without modifying the permeability and other properties of the hydrogel, as it can increase the stiffness of the hydrogel through interconnecting the collagen fibers.^[^ ^150^ ^]^ Through these platforms it was demonstrated that differences in the hydrogel stiffness could alter the phenotype of liver cancer cells from malignant (in compliant hydrogels gels) to a suppressed‐malignant (in stiffer hydrogels) (Figure 5i–j).^[^ ^150^ ^]^

This approach can also be used for studying the effect of hydrogel degradation without affecting matrix permeability. By incubating the hydrogel in MMP‐1 the elastic modulus was reduced from 4 to 0.5 kPa, this modification of the mechanical properties promoted cancer cells proliferation, and at the same time led to a reduced expression of E‐cadherin and lower detoxification capacity.^[^ ^151^ ^]^

Such examples evidence that Col I has been extensively explored in the field of 3D in vitro tumor modeling, and its processing into different platforms that differ in properties like stiffness, architecture, bioactivity, or degradation, by combining or crosslinking it with biomaterials. Nevertheless, it should be considered that ECM is constantly remodeled during tumor progression. Indeed, in most solid tumors there is an increase in the expression of Col I, and increase in the activity of enzymes such as lysyl oxidase (LOX) and MMP, which are responsible for degradation and ECM crosslinking, respectively. Therefore, the design of dynamic hydrogels that could modulate the biomechanical and chemical properties over time (4D hydrogels) could recreate the TME changes over different disease stages. The introduction of post‐gelation modifications in hydrogels by click chemistry or enzymes^[^ ^181^ ^]^ or the use of stimuli‐sensitive biomaterials^[^ ^182^ ^]^ to obtain new dynamic 4D hydrogels is starting to be explored in the field of tissue engineering, and this knowledge could be applicable to the development of more biomimetic 3D in vitro models. Another aspect that should be explored is the source of Col I and the combination of different collagen types. In general, collagen of rat origin is used for manufacturing these models, and it should be explored if this collagen source might have any impact in the generated data. Adding to this, the tumor ECM is a complex collagen mixture, among other components, and the use of only Col I might not be representative enough of the full TME complexity and matrix components. The effect of introducing other collagens in the hydrogel matrix should be investigated, as the phenotype and behavior of cancer cells might be completely different.^[^ ^148^ ^]^

### 4.2 Gelatin Hydrogels

Although collagen has been widely used for creating bioengineered TME's, it encompasses several limitations including the difficulty in tailoring Col I hydrogels properties such as the degree of crosslinking, degradation rate, the introduction of other biomolecules, or its manipulation. Gelatin is a natural‐origin biomaterial obtained from the denaturation of collagen.^[^ ^191^ ^]^ Similarly to collagen, gelatin has integrin binding motifs, such as RGD, for cell adhesion, and degradable sites recognized by MMPs,^[^ ^191^ ^]^ being susceptible to enzymatic degradation. Gelatin undergoes a thermoresponsive gelation below 30–35 °C through the establishment of noncovalent interactions.^[^ ^192^ ^]^ The thermo‐responsive behavior of gelatin has enabled its use as a sacrificial micromolding material, which allows the fabrication of microwells in hydrogels where 3D spheroids of cancer cells or tumor/stromal cells can be formed.^[^ ^193^, ^194^ ^]^ However, the main limitations, apart from its gelation below physiological temperatures, is that this biomaterial requires a high concentrations to generate hydrogels with appropriate mechanical properties.^[^ ^195^ ^]^ However, the mechanical properties of gelatin can be improved by crosslinking via chemical and/or enzymatic methods. Indeed, crosslinked gelatin hydrogels have demonstrated to be excellent ECM‐mimetic platforms for 3D cell culture. **Table** **2** summarizes the most relevant hydrogels used for cancer in vitro modeling using gelatin and its hybrid derivatives.

**Table 2:** 3D In vitro cancer models established in gelatin and gelatin‐biomaterial hybrid hydrogels. Abbreviations: cancer‐associated fibroblasts (CAFs), collagen I (Col I), extracellular matrix (ECM), epidermal growth factor receptor (EGFR), epithelial‐mesenchymal transition (EMT), glioblastoma multiform (GBM), gelatin‐methacrylamide (GelMA), gelatin‐norbornene (GelNB), glycine (Gly), high throughput screening (HTS), horseradish peroxidase (HRP), human bone marrow‐derived mesenchymal/stromal stem cells (hBM‐MSC), human mammary fibroblasts (HMFs), human umbilical vein cells (HUVEC), hyaluronic acid (HA), hyaluronic acid‐methacrylate (HA‐MA), hypoxia induced factors 1*α* (HIF‐1*α*), insulin‐like growth factor (IGF), lysyl oxidase (LOX), matrix metallopeptidase (MMP), nanoparticles (NPs), normal human lung fibroblasts (NHLFs), polyethylene glycol (PEG), PEG‐diacrylate (PEGDA), polyvinyl alcohol (PVA), transglutaminase (TG), and tyrosine (Tyr)

3D In vitro tumor model	Hydrogel composition	Model cells	Application	Achievements	Ref.
Breast cancer	GelMA	MDA‐MB‐231	Cell growth and drug screening	3D spheroids formed in GelMA hydrogels (stiffness: 4.81 kPa) displayed stemness, higher invasiveness, tumorigenic phenotype and higher resistance to paclitaxel than their 2D counterparts.	^[^ ^200^ ^]^
	GelMA	MDA‐MB‐231/MCF‐7/MCF10A	Invasion and migration	Microengineered hydrogels produced by photolithography containing circular constructs containing breast cancer cells dispersed in GelMA hydrogels of ≈750 Pa, embedded in a low stiffness GelMA hydrogel (less than 400 Pa). OnlyMDA‐MB‐231 metastatic cells invaded the surrounding hydrogel, whereas MCF‐7 or MCF‐10A formed noninvasive 3D spheroids.	^[^ ^201^ ^]^
	GelMA	HUVEC/MCF‐7	Angiogenesis	Microfluidic device where multicellular 3D spheroids are embedded in the hydrogel. Cancer cells do not exhibit migration outside the 3D spheroid, but endothelial cells migrated to 3D spheroids periphery. High doxorubicin doses were required to penetrate the formed microtumors and their size was reduced only after 3 days of incubation with the chemotherapeutics.	^[^ ^114^ ^]^
	GelMA	HUVECs/MCF7/MDA‐MB‐231/THP‐1/TALL‐1	TME effect on T cell recruitment	TOC formed by a bilayered hydrogel with an inner layer monocytes and breast tumor spheroids and an outer layer with HUVECs cells, where T cells can be perfused. Monocyte inclusion promoted T cells extravasation cytokine‐mediated.	^[^ ^205^ ^]^
	Gelatin crosslinked by TG	HMF	Effect of the stiffness	Hydrogels with stiffness of 200 (compliant), 300 (moderate) and 1100 Pa (stiff) were produced. HMFs proliferated in all the hydrogels, but proliferated more in moderate stiff gels. HMFs exhibited a spindle‐like in compliant and moderate hydrogels, whereas in stiff hydrogels exhibited a rounded morphology with protrusions.	^[^ ^206^ ^]^
	Gelatin + alginate	MDA‐MB‐231	Spheroid production	3D printed hydrogel for 3D spheroids generation. Bioink with higher gelatin and lower alginate content enable the production of larger 3D spheroids, due to the lower stiffness (7.92 kPa).	^[^ ^225^ ^]^
	Gel + alginate	MDA‐MB‐231/IMR‐90	Invasion and migration	3D bioprinted constructs with cancer cells in the core and fibroblast are in the outer part, separated with an acellular hydrogel. Cancer cells form spheroids and fibroblast migrate toward the tumor side, infiltrating into the tumor spheroids.	^[^ ^224^ ^]^
	GelMA + Col I	MDA‐MB‐231	Effect of the stiffness	Increases in stiffness from 2 to 12 kPa, change from proteolytically independent to a proteolytically dependent invasion behavior through a MENA‐related pathway.	^[^ ^216^ ^]^
	GelMA + Col I	MCF7	Drugs and NPs diffusion	Microfluidic device consisting in a central channel seeded with cancer cells laden in hydrogel mimicking the tumor matrix (6 kPa) or healthy (0.15 kPa) tissue ECM and two capillary‐like lateral channels. Molecules and NPs can diffuse to the central channel, being lower in the case of tumor mimicking ECM.	^[^ ^230^ ^]^
	GelMA + PEGDA	MDA‐MB‐231	Invasion and migration	Metastatic breast cancer cells exhibited a more invasive phenotype with a spindle‐shaped morphology in GelMA dominant hydrogels (1 kPa) in comparison to PEGDA predominant hydrogels (8 kPa), due to a lower stiffness and higher degradability.	^[^ ^211^ ^]^
	GelMA + 4‐arm‐PEG‐ acrylate‐RGD	3T3‐L1 differentiated to adipocytes/HCC1806/MDA‐MB‐231	Effect of stiffness	Microwell array system on hydrogels with stiffness ranging from 200 Pa (healthy tissue) or 3 kPa (tumor tissue). The hydrogels were laden with preadipocytes and cancer cells. Preadipocyte stromal cells differentiation and maturation was affected by the stiffness. At stiffness similar to that of the tumor tissue adipogenesis was inhibited.	^[^ ^214^ ^]^
	GelMa + 4‐arm‐PEG	HCC1806/primary mammary organoid	Spheroid formation	Microwell arrays system with different stiffness enable the spheroid formation, small differences in stiffness (460 vs 600 Pa) did promoted differences in 3D spheroids growth.	^[^ ^231^ ^]^
	GelMA + HA‐MA	21PT/21MT‐2	Hypoxia	Hydrogels with a stiffness of 1.16 kPa were useful to study hypoxia. Hypoxia decreased 3D spheroids size and promoted EMT, cell migration, and increased LOX secretion. LOX inhibitors reduced cells viability, EMT and migration.	^[^ ^232^ ^]^
Breast cancer metastasis to bone	GelMA + hydroxyapatite	Fetal osteoblasts/hBM‐MSCs/MDA‐MB‐231	Metastasis	3D printed hydrogels laden with bone stroma and breast cancer cells seeded on top. Cancer cells proliferation was increased by osteoblasts and hBM‐MSCs presence. Osteoblast and hBM‐MSCs proliferation and alkaline phosphatase activity were reduced by cancer cells.	^[^ ^202^ ^]^
Prostate cancer metastasis to bone	GelMa + HA‐MA	PC‐3/human osteoblasts	HTS	3D Microgels produced by superhydrophobic surfaces technology. Osteoblast mineralized the hydrogel mimicking the bone microenvironment. Cancer cells were more resistant in the 3D microgels than when cultured in scaffold‐free 3D spheroids.	^[^ ^220^ ^]^
Osteosarcoma	GelMA + Matrigel	MG‐63	Drug screening	Liquid overlay technique to produce reproducible 3D microtumor spheroids. The microgel 3D spheroids have a more invasive phenotype and a higher resistance to lorlatinib than cells cultured in 3D hydrogels.	^[^ ^93^ ^]^
	GelMA + PEGDA	MG63/hFOB1.19	Effect of stiffness and adhesion molecules	Stiffness regulated osteosarcoma cells proliferation by integrin‐mediated focal adhesion, meanwhile the adhesion molecules density (provided by gelatin) regulated osteoblasts proliferation by an integrin‐mediated adherents junction pathway.	^[^ ^212^ ^]^
	Ferulic acid gelatin	Primary osteosarcoma cells	Hypoxia	Generation of a hypoxia gradient within the hydrogel. O_2_ worked as a physicotactic agent, showing that hypoxia guided cell invasion through HIF‐1*α*.	^[^ ^209^ ^]^
Hepatic cancer	Col beads + Gelatin‐thiol + 4‐arm‐PEG‐thiol + HRP + Tyr‐Gly	NIH3T3/HUVEC/HepG2	Vascularized microtumors	Microtumors comprising 3D spheroids, endothelial cells and collagen microparticles, encapsulated within a fibroblast cell sheet. Endothelial cells formed capillary‐like structures, and hepatocytes function was higher than cells aggregates without the cell layer coating.	^[^ ^215^ ^]^
	GelNB + PEG‐4‐arm‐thiol	Huh‐7	Effect of the stiffness	Lower stiffness and greater gelatin content improved cells metabolic activity, but had no effect in hepatocyte specific cellular functions. When heparin was introduced in the hydrogel, it enabled to sequester and release hepatocyte growth factor, improving the CYP450 activity and urea secretion.	^[^ ^213^ ^]^
	Gelatin + PVA	HepG2	Invasion and migration	HepG2 cells formed large cellular aggregates with different morphologies. Frontline cells exhibited lamellipodia‐like structures.	^[^ ^125^ ^]^
GBM	GelMA	U373	Spheroids formation	Spheroids and cells encapsulated in the gel upregulated pro‐survival and MMP genes and reduced the expression of apoptosis‐activating genes.	^[^ ^203^ ^]^
	GelMA	HUVEC/U87	Angiogenesis	Hydrogel microwells recapitulating angiogenesis. Cancer cells formed 3D spheroids inside the wells, meanwhile HUVECs were seeded in 2D outside the wells. Cancer cells promoted the invasion of endothelial cells inside the hydrogel and the formation of tubular‐like structures.	^[^ ^204^ ^]^
	GelMA + HA‐MA	U251	Invasion and migration	GBM invasion is enhanced in softer hydrogels (8.8 kPa) and reduced in the presence of HA of 60 kDa. In the absence of HA in the hydrogel, GBM cells synthetize it to stimulate invasion.	^[^ ^233^ ^]^
	GelMA + HA‐MA	U87MG	Platform	Platform containing hydrogels with spatial gradients of crosslinking, cell density and HA content.	^[^ ^217^ ^]^
	GelMA + HA‐MA	Patient derived GBM	Invasion and migration	Cells invasiveness depends of the molecular weight of HA, having a more invasive phenotype when HA of 10 or 60 kDa was immobilized than with 500 kDa HA.	^[^ ^234^ ^]^
	GelMA + HA‐MA	U87MG/U87 EGFR+	Effect of HA	The presence of HA of 1630 kDa promoted cell clustering and the expression of malignancy‐associated genes in the range of 0.3–0.5% w/v (above this content HA presence has a negative effect). There is a connection between CD44 and EGFR in the cell malignancy.	^[^ ^235^ ^]^
	GelMA + HA‐MA	U87	Invasion and migration	Platform with hypoxia gradients. Cancer cells adapted to the hypoxic environment by switching to a more malignant phenotype (activating ERK), becoming more invasive, overexpressing MMPs and secreting HA.	^[^ ^236^ ^]^
	HA‐MA + GelMA	NHLFs/HUVEC/U87‐MG	Stroma interactions	Upregulation of genes related with angiogenesis, ECM remodeling enzymes, GBM malignancy (MGMT, EGFR, PI3K‐Akt, Ras/MAPK). Higher resistance to TMZ in comparison with monoculture hydrogels.	^[^ ^219^ ^]^
	GelMA + HA‐MA	Patient derived GBM cells with EGFR mutations	Effect of ECM in drug response.	The effect of erlotinib on GBM cells cultured on the hydrogel was dependent on the EGFR mutation. Erlotinib had no cytotoxic effect in EGFRvIII cells. However, cells with EGFR + were only susceptible to the drug in the absence of HA, suggesting that HA promotes the inhibition of this receptor by deactivating STAT3.	^[^ ^218^ ^]^
	HA‐MA + GelMA + VEGF immobilized	NHLFs, HUVECs and U87‐MG	Angiogenesis	Recreate the tumor vasculature in a hydrogel platform. GBM cells produced the endothelial network regression in endothelial cells.	^[^ ^221^ ^]^
	GelMA + PEGDA	U87MG	Hypoxia	The high crosslinking achieved by the degree of substitution, the GelMA concentration, the size of the hydrogel and PEGDA presence enable the establishment of a hypoxia gradient within the matrix, that stimulate the expression of HIF‐1, VEGF and MMP.	^[^ ^210^ ^]^
	Gel + HA (HyStem)	U‐87 MG, Primary human GBM and astrocytes	Drug screening	Non‐tumor astrocytes in contact with GBM cells promoted temozolomide, clomipramine and vincristine resistance. Astrocytes transfer mitochondria through tunneling nanotubes to rescue the damaged cancer cells. The presence of HA promoted nanotubes formation.	^[^ ^237^ ^]^
	Gel + HA (HyStem)	U‐87 MG/SNB‐19/Primary human GBM	Stroma interactions	The coculture of microglia cells has a positive effect in GBM migration and proliferation, and reduced the efficacy of temozolomide, clomipramine and vincristine.	^[^ ^238^ ^]^
	Gelatin + TG + PEG4000	U87 HUVEC	Endothelial molecules diffusion into tumors	Microfluidic device. Cancer cells were dispersed in the hydrogel, which contained a lumen where endothelial cells were culture to simulate a blood vessel structure. Antioxidant molecules were tested in this model, showing a reduction in ROS and increase in GSH.	^[^ ^113^ ^]^
	Gelatin + fibrinogen + alginate	SU3/U87	Drug screening and stemness	3D printed hydrogel where glioma stem cells maintained stemness markers (Nestin), had differentiation potential (glial fibrillary acidic protein and *β*‐tubulin III) and expressed VEGF. Higher resistance to temozolomide than 2D models.	^[^ ^227^ ^]^
Lung cancer	Gelatin + alginate	A549/95‐D	Invasion and migration	3D printed model where cancer cells have an enhanced cell invasion and migration behavior compared with 2D models.	^[^ ^223^ ^]^
	Gelatin + alginate	Patient‐derived nonsmall cell lung cancer/lung CAFs	Stroma interactions	3D printed model where cells formed large spheroids with higher vimentin and *α*‐SMA expression in cocultures, confirming the crosstalk between cells.	^[^ ^239^ ^]^
Cervical tumor model	Gelatin + alginate + Matrigel	HeLa	EMT modeling and evaluation	3D printed hydrogel to study the EMT promoted by TGF‐*β*. Cells formed 3D spheroids that in presence of TGF‐*β* changed the morphology to spindle‐like, downregulated E‐cadherin, and upregulated mesenchymal markers. EMT was inhibited by disulfiram and C19 (EMT pathway inhibitor C19).	^[^ ^228^ ^]^
Ovarian cancer	GelMA	OV‐MZ‐6	Spheroid formation	Cells formed spheroids that resembled ascites, and their proliferation and size depend on matrix degradation.	^[^ ^199^ ^]^
Pancreatic ductal adenocarcinoma	GelNB + thiolated HA	COLO‐357	Effect of stiffness	Cells formed spheroids, and stiffer hydrogel promoted the formation of smaller spheroids with an upregulation of SHH and MMP‐14.	^[^ ^222^ ^]^
	GelNB‐hydroxyphenyl‐acetic acid + PEG4SH or thiolated‐HA + tyrosinase	COLO‐357	Effect of stiffness	The presence of HA or the enzymatic reduced the cell growth, and induced a more invasive phenotype (EMT).	^[^ ^240^ ^]^
Melanoma	GelMA	Sk‐MEL28/WM35	Interaction IGF and ECM	Interactions between IGF and ECM have an effect in melanoma progression. Peptide antagonists that inhibits the binding of IGF‐I: IGF binding proteins3 to vitronectin, reduced cell growth and invasion.	^[^ ^241^ ^]^
Colorectal	Gelatin–phenol crosslinked with HRP	Patient xenograft	Effect of stiffness	Hydrogels with stiffness similar to the normal (2.6 kPa) or malignant (34 kPa) human colon enabled the formation of 3D organoids from colorectal cancer. Higher stiffness increased organoid growth, metabolism, and hypoxia comparable to Geltrex.	^[^ ^207^ ^]^

#### 4.2.1 Gelatin Methacrylamide (GelMA)

MA groups can be crosslinked by ultraviolet (UV)/vis light in presence of a photoinitiator, yielding inexpensive and cytocompatible hydrogels. Cytocompatible photoinitiators such as Irgacure 2959 or lithium phenyl‐2,4,6‐trimethylbenzoylphosphinate (LAP), among others, can be used to crosslink GelMA within seconds to few minutes. The stiffness and biomechanical properties of the resulting gel is dependent on the exposure time, the degree of substitution, addition of nanobiomaterials, and photoinitiator concentration,^[^ ^196^, ^197^, ^198^ ^]^ to adapt them to the TME. For instance, with polymer concentrations ranging from 2.5% to 7% w/v, stiffness between 0.5 to 9.0 kPa can be achieved, without affecting hydrogels diffusional properties.^[^ ^199^ ^]^ Moreover, GelMA hydrogels have a high transparency, enabling microscopic visualization of cells. Several previous studies have proved the suitability of GelMA for culturing cancer cells and modeling several types of malignancies such as breast cancer^[^ ^200^, ^201^, ^202^ ^]^ or GBM.^[^ ^203^, ^204^ ^]^

GelMA hydrogels have proved their potential for developing 3D platforms for investigating the invasiveness and chemotherapeutics‐responsiveness of breast cancer cells. GelMA hydrogels with stiffness of 4.8 kPa sustained the formation of MDA‐MB‐231 spheroids, showing an upregulation of genes associated with stemness. After 5 days in culture, cancer cells started to migrate out of the 3D spheroids mass, exhibiting an upregulation of genes related with breast cancer invasiveness, when compared to those obtained with standard scaffold‐free 3D spheroids generated in low‐attachment conditions (liquid overlay technique). Interestingly, cells cultured in gelatin hydrogels were intravenously injected into mice, and after 6 weeks tumor nodules could be found in lungs and in the thoracic cavity, a factor that was not observed for tumors generated from cells cultured in 2D monolayers.^[^ ^200^ ^]^ All these findings suggested that GelMA matrices are more appropriate to recapitulate the tumorigenic potential and invasiveness of breast cancer cells when compared to 2D, and also scaffold‐free 3D spheroids. In addition, these tumor constructs showed a higher resistance to taxane drugs, even upregulating the expression of breast chemoresistance genes.^[^ ^200^ ^]^ In another interesting approach, researchers prepared a microengineered hydrogel of GelMA using a two‐step photolithography‐based method. This approach enabled the fabrication of circular constructs containing breast cancer cells dispersed in GelMA hydrogels of ≈750 Pa, embedded in a low stiffness GelMA hydrogel (less than 400 Pa).^[^ ^201^ ^]^ This platform was then used for evaluating the invasive potential of different types of breast cancer cells: i) metastatic cells MDA‐MB‐231, ii) nonmetastatic (MCF‐7), and iii) nonmalignant cells (MCF10A). Only the metastatic cells were able to invade the surrounding hydrogel, meanwhile the other cell types formed 3D clusters and remained in the original well. This platform can be interesting for evaluating the effect of stiffness in cancer cells phenotype in a HTS model. GelMA can also be combined with nanocrystalline hydroxyapatite to recapitulate the metastatic bone environment in breast cancer, a tissue that is a preferential metastization site for this type of malignancy.^[^ ^202^ ^]^

**Figure 6:**
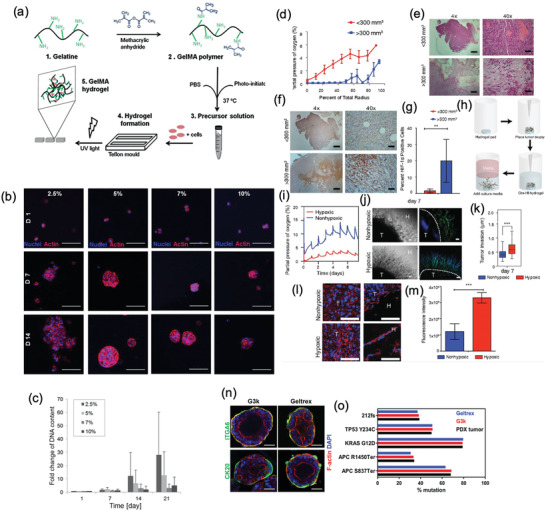
Gelatin‐based hydrogels for 3D in vitro tumor modeling. a–c) Hydrogels of GelMA for the fabrication of tumor models of ovarian cancer. a) Fabrication of GelMA hydrogels. Gelatin is functionalized with methacrylic anhydride to form GelMA. Then, the polymer is dissolved in PBS in the presence of a photoinitiator and cells at 37 °C, and photo crosslinked with ultraviolet light. b,c) Morphology and cellular proliferation of OV‐MZ‐6 spheroids in GelMA hydrogels of 2.5% (0.7 kPa), 5% (3.4 kPa), 7% (7.3 kPa), and 10% (16.5 kPa). b) Confocal images showing the nuclei in blue and in red the cytoskeleton (scale bar: 100 µm). c) DNA content at days 1,7, and 21 days normalized to day 1. The cellular morphology depends on hydrogel stiffness, at lower concentrations cells form lose aggregates and at higher concentrations cells form small 3D spheroids. Moreover, cells proliferate faster at lower concentrations. Reproduced with permission.^[^ ^199^ ^]^ Copyright 2014, Elsevier. d–m) Model of sarcoma cells invasion in hypoxic hydrogels of FA‐Gel. d) Dissolved O_2_ in murine sarcoma cells from murine tumors. e,f) Hematoxylin and eosin (e) and HIF‐1 (f) stainings in small and large tumors (scale bars: 200 µm, 4× and 20 µm, 40×). g) Expression of HIF‐1 in tumors. h) Scheme of the encapsulation of the tumor biopsy in the FA‐Gel hydrogels. i) Dissolved O_2_ in hydrogels in hypoxic and nonhypoxic conditions over time. j) Sarcoma tumors in the hydrogels under light microscopy and fluorescence microscopy. Actin in green and nuclei in blue (scale bars: 100 µm.). k) Tumor invasion within the hydrogel under hypoxic and nonhypoxic conditions. l,m) Collagen deposition in tumor after 7 days in culture. l) Immunofluorescence images. Collagen in red, nuclei in blue (scale bars: 25 µm). m) Quantification of the collagen (^**^ *p* < 0.01, ^***^ *p* < 0.001). Reproduced with permission.^[^ ^209^ ^]^ Copyright 2014, National Academy of Sciences. n,o) Hydrogels of gelatin–phenol enzymatically crosslinked for the culture of colorectal organoids. n) Organoids after 15 days of culture, sowing basolateral expression of ITGA6 and CK20 and apical F‐actin expression, maintaining the epithelial polarity, in the hydrogel and Geltrex (scale bars: 50 µm). o) Profiles of mutations in organoids grown in the hydrogels or Geltrex for 9 days or the tumor xenograft after 9 days. Reproduced with permission.^[^ ^207^ ^]^ Copyright 2019, Elsevier.

Another important use of GelMA hydrogels is for modeling ovarian cancer. GelMA hydrogels with stiffness of 3.4 kPa were suitable for the formation of 3D spheroids comprising human epithelial ovarian cancer cell line OV‐MZ‐6, an elegant model that resembled the ascites formed in the abdominal cavity after metastasis (**Figure** **6**a–c). The addition of tumor‐ECM components including laminin‐411 and HA into the hydrogel network increased the formation of 3D spheroids and cellular proliferation. Interestingly, when MMP‐mediated degradation was inhibited, cells were not able to degrade the hydrogel, exhibiting a reduced proliferation and 3D spheroids formation within the matrix.^[^ ^199^ ^]^

GBM cells were also cultured in GelMA hydrogels crosslinked with visible light using eosin Y as photoinitiator, triethanolamine as electron donor, and *N*‐vinyl‐2‐pyrrolidinone as catalyst.^[^ ^203^ ^]^ In this platform, U373 cells exhibited an increased expression of Bcl‐2 (associated with pro‐survival), as well as MMP‐associated genes, and displayed a reduced expression of apoptosis‐activating genes.^[^ ^203^ ^]^

GelMA hydrogels can be also used for studying the effect of the immune systems in the tumor progression using microfluidic devices namely to study the effect of the TME in T cells recruitment.^[^ ^205^ ^]^ In this approach researchers fabricated a microdevice containing a bi‐layered GelMA hydrogel with 6.5 kPa stiffness thought photopatterning. Monocytes (THP‐1) and breast tumor spheroids (MDA‐MB‐231 and MCF‐7) were seeded in the inner side of the hydrogel and in the outer layer endothelial cells (HUVEC) were included. After 4–6 days of culture, T cells (TALL‐1) were perfused into the chip. In this set up, monocytes promoted the extravasation of T cells due to an increase of CCL4, CCL5, CCL11, CXCL8, and CCL20, that could be related to hypoxia. Interestingly, TALL‐1 does not have receptors for CXCL8, but it could have an impact in the endothelial barrier permeability, increasing the extravasation of T cells.^[^ ^205^ ^]^

#### 4.2.2 Enzymatically Crosslinked Hydrogels

Another relatively underexplored strategy for preparing gelatin hydrogels for cancer modeling is to use enzymes to crosslink the functional groups of gelatin. Lysine and glutamine residues of gelatin can be crosslinked using transglutaminases (TG), obtaining hydrogels with low stiffness but high stability.^[^ ^206^ ^]^ By increasing the TG concentration, it is possible to increase hydrogels stiffness up to 1.1 kPa.^[^ ^206^ ^]^ Gelatin–phenol can also be crosslinked enzymatically by horseradish peroxidase (HRP) in the presence of H_2_O_2_, and the hydrogel stiffness is easily tunable. Ng et al. showed that they could prepare hydrogels with stiffness of the normal or tumor human colon, and growth organoids from colorectal cancer inside (Figure 6n,o). Higher stiffness increased the organoid growth, metabolism, and hypoxia, comparable to Geltrex.^[^ ^207^ ^]^

It is also relevant to highlight the use of ferulic acid‐gelatin (FA‐Gel) hydrogels for creating hypoxia models, which is essential to study its effect in tumor growth and cellular response, as frequently tumors present hypoxia gradients. FA‐Gel can be crosslinked via the enzyme laccase, which reacts with the FA group to form di‐FA with O_2_ consumption.^[^ ^208^ ^]^ The stiffness and degradability of these hydrogels can be readily tuned by altering the polymer and enzyme concentration.^[^ ^208^ ^]^ This platform was used to evaluate the impact of hypoxia gradients in a sarcoma model. These researchers encapsulated primary osteosarcoma cells in the 3D hydrogels and recapitulated the hypoxic gradient occurring in vivo, discovering that in hypoxic conditions cancer cells have a more invasive behavior mediated by HIF‐1*α* expression, and that O_2_ was acting as a physicotactic agent (Figure 6d–m).^[^ ^209^ ^]^

#### 4.2.3 Hybrid Gelatin Hydrogels

One of the most used polymers that have been combined with gelatin is the inert PEG or its derivatives. PEG‐diacrylate (PEGDA) has been combined with GelMA as a strategy to reduce the degradation rate of the hydrogel and also to limit the diffusion of molecules above 40 kDa.^[^ ^210^ ^]^ These hydrogels are suitable for studying the effect of hypoxia in cancer cells.^[^ ^210^ ^]^ The high crosslinking achieved by tuning GelMA degree of substitution and concentration, the size of the hydrogel and PEGDA presence enabled the establishment of an hypoxia gradient within the matrix.^[^ ^210^ ^]^ PEGMA/GelMA hydrogels can also be used to evaluate the effect of matrix degradability in the invasiveness of metastatic cells. Metastatic breast cancer cells (MDA‐MB‐231) cultured in GelMA/PEGDA hydrogels exhibited an invasive phenotype with a spindle‐shaped morphology in GelMA rich hydrogels, due to the softer and more degradable matrix, whereas in hydrogels where PEGDA was predominant, cells formed 3D spheroids.^[^ ^211^ ^]^ Another important use of PEGDA in GelMA hydrogels is to add an inert material to the hydrogel to study the effect of stiffness and adhesion ligand densities in bone tumor model.^[^ ^212^ ^]^ These hydrogels were explored for investigating the effect of hydrogel stiffness in osteosarcoma cells proliferation in 3D by evaluating integrin‐mediated focal adhesions. Interestingly, adhesion molecules density (provided by gelatin) regulated osteoblasts proliferation by an integrin‐mediated adherents junction pathway.^[^ ^212^ ^]^

**Figure 7:**
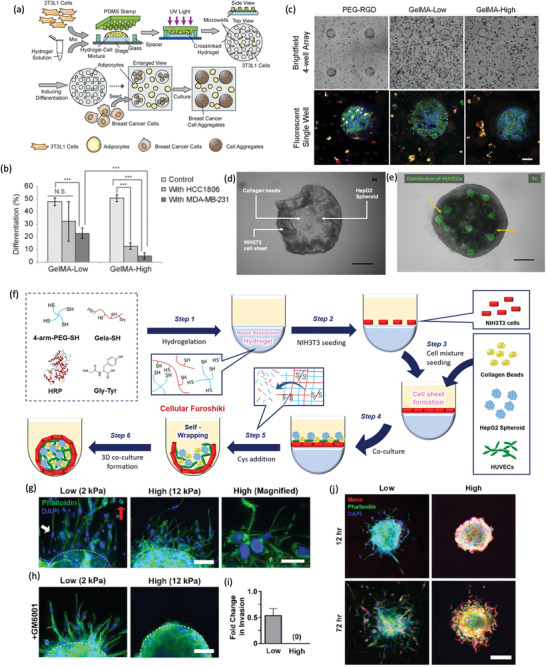
Hybrid gelatin hydrogels formulated by combination with collagen and PEG to generate bioactive platforms for in vitro cancer modeling. a–c) Microwell platform to study the effect of the stiffness in the cross‐talk of adipocytes and breast cancer cells. Microwells were produced in GelMA and 4‐arm polyethylene glycol acrylate‐RGD hydrogels with stiffness ranging from 200 Pa (healthy tissue) or 3 kPa (tumor tissue). Mouse embryo fibroblasts (3T3‐L1) differentiated to adipocytes were dispersed in the hydrogel and breast cancer cells (HCC1806 cells and MDA‐MB‐231) into the microwells. a) Scheme of the fabrication of the microwell system. b) Stromal cell‐laden hydrogel‐based microwell arrays for culturing human breast cancer cell line HCC1806. Brightfield and immunofluorescence in the microwells (blue nuclei, red adipocytes, and green E‐cadherin) (scale bars: 500 µm, 4‐well arrays, and 100 µm, single well). c) Adipocyte differentiation efficiency in cocultures with HCC1806 spheroids or MDA‐MB‐231 spheroids. ^***^ *p* < 0.0001, N.S.: no significant differences. Reproduced with permission.^[^ ^214^ ^]^ Copyright 2018, Elsevier. d–f) Cell microstructures made of HepG2, HUVECs, NIH3T3 cell sheet and collagen beads. d to Wrapped microstructures at day 1. White arrows show the different components. e) CD31 staining (green) at day 7 showing HUVEC connected between themselves (scale bars: 500 µm). f) Scheme of the cellular microstructures fabrication. NIH3T3 were seeded on top of a stimuli‐sensitive hydrogel. Then, cells and collagen beads were placed on the cell sheet. After the hydrogel degradation, the microstructures are formed. Reproduced with permission.^[^ ^215^ ^]^ Copyright 2020, Springer Nature. g–j) Cell invasion of MDA‐MB‐231 in GelMA and Col I hydrogels. g) MDA‐MD‐231 invasion in hydrogels of 2 and 12 kPa. Marked with white arrow elongated cells and with red arrow the ameboid cells (scale bar = 50 µm, left and 25 µm, right). h,i) Cell invasion of MDA‐MB‐231 in presence of a pan‐MMP inhibitor (GM6001), which had no effect in 2 kPA hydrogels, but eliminated the actin‐enriched protrusions in hydrogels with higher stiffness (scale bar = 50 µm). Green channel: cytoskeleton and blue channel: nuclei. j) Immunofluorescence micrographs of 3D spheroids showing the upregulation of MENA (scale bar = 150 µm) in low and high stiffness hydrogels. Reproduced with permission.^[^ ^216^ ^]^ Copyright 2020, Elsevier.

Another alternative for the crosslinking of gelatin and PEG hydrogels is the use of 4‐arm PEG chemically active derivatives. This approach was used to crosslink gelatin‐norbornene (GelNB) derivatives with PEG‐4‐arm‐thiol via UV light, generating hydrogels that were appropriate for culturing hepatocellular carcinoma cells (Huh‐7). The stiffness of the resulting hydrogel was tuned by altering the degree of substitution and the polymer concentration, lower stiffness and a higher gelatin content improved cells metabolic activity, but did not had any effect in hepatocyte‐specific cellular functions. Interestingly, the incorporation of heparin into the GelNB matrix improved CYP450 activity and urea secretion, as well as enabled the sequestration and release of hepatocyte growth factor.^[^ ^213^ ^]^ In a different report, a similar formulation was used to evaluate the effect of stiffness on the cross‐interaction of cancer cells and adipocytes (**Figure** **7**a–c). For this purpose, GelMA and 4‐arm PEG‐acrylate‐RGD hydrogels were used to fabricate a microwell array by micromolding techniques. Through this technology researchers formulated GELMA/PEG hydrogels with different stiffness 200 Pa (healthy tissue) or 3 kPa (tumor tissue), and bioencapsulated mouse embryo fibroblasts (3T3‐L1) that were differentiated to adipocytes when laden within the hydrogel network. Upon seeding of human triple negative breast cancer cells (HCC1806 cells and MDA‐MB‐231) into the microwells it was discovered that preadipocyte stromal cells differentiation and maturation was significantly affected by substrate stiffness, and at stiffnesses similar to those of the original tumor tissue the adipogenesis was inhibited.^[^ ^214^ ^]^

Another interesting platform based on hybrid gelatin/PEG microgels was developed by crosslinking gelatin‐SH and 4‐arm‐PEG‐SH redox responsive hydrogel that was crosslinked via Gly‐tyrosine (Tyr) and HRP, were fibroblasts could proliferate, forming a layer on top of the hydrogel (Figure 7d–f). Then, researchers encapsulated human hepatocellular carcinoma (HepG2) spheroids, endothelial cells, and collagen microparticles with the formed fibroblast cell sheet, by just degrading the hydrogel. Endothelial cells were able to grow on top of the collagen microparticles, forming vessel‐like structures (Figure 7e). The incorporation of endothelial cells in the model, increased the cellular viability of the wrapped MTs, suggesting their important role of recreating the vasculature for the viability of HepG2 cells. Collagen beads also have an effect in the viability of cells, by creating spaces for nutrients and oxygen diffusion. Moreover, these MTs improved the cellular function of hepatocytes when compared with cellular aggregates without the cell layer coating it.^[^ ^215^ ^]^

Collagen has also been combined with gelatin for 3D in vitro tumor models generation. This combination has been employed for studying the effect of matrix stiffness in cancer cells invasiveness. Berger et al. prepared hydrogels of GelMA/Col I of low (2 kPa) and high (12 kPa) stiffness to investigate the invasion of metastatic breast cancer cells (MDA‐MB‐231) (Figure 7g–j). It was observed that cells cultured in 12 kPa hydrogels exhibited more actin‐enriched protrusions and a higher expression of MENA (an invadopodia protein related with metastasis through ECM remodeling), in comparison to that obtained in low stiffness matrices. Although hydrogel stiffness may delay cancer cells invasion, it promoted a change from proteolytically independent to a proteolytically dependent invasion behavior. This MENA‐related behavior was not observed in GelMA hydrogels without Col I, proving that the matrix composition is a critical factor that must be taken into consideration during the establishment of 3D in vitro models.^[^ ^216^ ^]^

**Figure 8:**
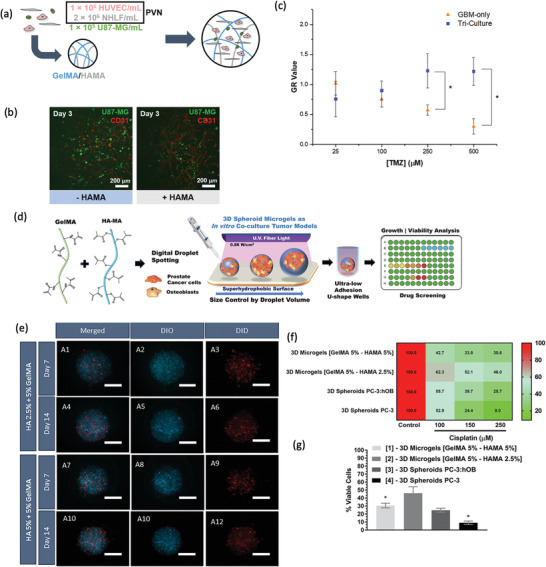
Gelatin hydrogels combined with hyaluronic acid or alginate for the fabrication of in vitro cancer models. a–c) Glioblastoma model in a hydrogel of GelMA and HA‐MA. a) Scheme of the coculture in perivascular cells and U87‐MC cells in hydrogels. b) Immunofluorescence of the hydrogel, in green U87‐MG cancer cells and in red CD31+ endothelial cells, being both in proximity (scale bar: 200 µm). c) Growth rate of inhibition (GR) at different concentrations of TM of the triculture model or only GBM cells (*p < 0.05). Reproduced with permission.^[^ ^219^ ^]^ Copyright 2019, Elsevier. d–g) Model of prostate cancer metastasis to bone using HAMA and GelMA beads. d) Scheme of the tumor model fabrication using superhydrophobic surfaces and its use. e) Fluorescence micrographs of hydrogel beads with different HAMA content containing prostate cancer cells (PC‐3) labeled with DiO (blue) and human osteoblasts labeled with DiD (red) (scale bar: 200 µm). f,g) Assessment of the cytotoxicity of cisplatin in microgel beads with different HA‐MA content and spheroids formed under low attachment conditions. f) Heat map representing the cell viability at increasing drug concentrations. g) Cell viability of each model incubated with 250 × 10^−6^ m of cisplatin. Reproduced with permission.^[^ ^220^ ^]^ Copyright 2019, Elsevier.

Another common polymer combined with gelatin is HA, especially for the creation of GBM models.^[^ ^217^, ^218^, ^219^ ^]^ Different approaches have been followed for the preparation of interpenetrated systems combining both polymers. One of the approaches followed is the photopolymerization of GelMA and HA‐methacrylate (HA‐MA) together.^[^ ^218^, ^219^, ^220^ ^]^ For example, GelMA/HA‐MA hydrogels functionalized with immobilized VEGF were generated for creating an in vitro model of GBM. By adjusting cells concentration (normal human lung fibroblasts (NHLFs), HUVECs and U87‐MG) and the ratio of HA and VEGF, the stiffness and vasculature of the TME was recapitulated.^[^ ^221^ ^]^ By using this strategy it was observed a close interaction of the vasculature and the cancer cells which promoted an upregulation of angiogenesis, ECM remodeling and GBM malignancy‐associated genes, and reduced the sensitivity to temozolomide (chemotherapeutic used in GBM) (**Figure** **8**a–c).^[^ ^219^ ^]^ GelMA and HA‐MA hydrogels were also used for recreating the bone environment for studying the prostate metastasis to bone. These microhydrogels were formulated by using superhydrophobic surfaces (Figure 8d).^[^ ^220^ ^]^ This technology enabled a rapid, in‐air production of cocultured cell‐laden GelMA/HA‐MA microgels. The size and cell density inside each microgel can be easily controlled by just digitally dispensing droplets of specific volume on the superhydrophobic surfaces which are HTS compatible. The produced microgels were used for establishing prostate cancer‐to‐bone metastasis model, via the coculture of prostate cancer cells (PC‐3) and human osteoblasts (hOB) (Figure 8d–g). 3D microgel cocultured osteoblasts produced calcium deposits in vitro, thus recapitulating the native bone TME to where PC‐3 cancer cells generally migrate to. 3D microgels were used drug screening, being observed that 3D microgels were more resistant to cisplatin than monoculture microgels or cocultured control scaffold‐free 3D spheroids (Figure 8f,g).^[^ ^220^ ^]^

The thiol−norbornene orthogonal click chemistry crosslinking can also be used to prepare hydrogels comprising HA and gelatin. Importantly, the reaction can occur with visible light using eosin‐Y as a photoinitiator, which reduces any potential cellular damage provoked by UV exposition, required when using other photoinitiators (i.e., Irgacure 2959). Using this approach researchers fabricated biomimetic hydrogels containing GelNB and thiolated‐HA, and tested those as a biomimetic matrix for pancreatic ductal adenocarcinoma cell growth. The researchers tested the effect of the stiffness of this hydrogel on the behavior of COLO357, and it was observed that stiffer hydrogels promote the formation of smaller 3D spheroids with an upregulation of SHH and MMP‐14 biomarkers.^[^ ^222^ ^]^

Gelatin has been combined with alginate to fabricate bioprintable hydrogels to be used as lung,^[^ ^223^ ^]^ brain,^[^ ^224^ ^]^ breast^[^ ^225^ ^]^ tumor models, among others. The inclusion of bioactive NPs in such systems could be used in the context of mineralized tissues.^[^ ^226^ ^]^ Dai et al. 3D bioprinted a mixture of alginate, gelatin, and fibrinogen, crosslinked by Ca^2+^, TG, and thrombin respectively to generate GBM models. Interestingly, in this matrix, glioma stem cells were able to maintain key stemness biomarkers (e.g., Nestin), and at the same time, cells exhibited some glial differentiation and increased VEGF secretion over time. Also, when GBM cells were 3D bioprinted in this biomaterial ink, a higher resistance to temozolomide was obtained, in comparison to that of 2D monolayered models.^[^ ^227^ ^]^ 3D bioprinted alginate/gelatin hydrogels can also be employed for evaluating the invasiveness of cancer cells.^[^ ^223^, ^224^, ^228^ ^]^ For instance, alginate/Matrigel/gelatin hydrogels were used to test the effect of transforming growth factor‐beta (TGF‐*β*)‐mediated HeLa cells migration, and when hydrogels were exposed to these molecules, cells forming 3D spheroids exhibited EMT and upregulated mesenchymal markers expression.^[^ ^228^ ^]^

Even though the easy chemical functionalization of gelatin, GelMA is still the most popular derivative used in cancer modeling. New crosslinking methods using click chemistry avoiding UV light should be explored as they could reduce the cell damaging provoked by the UV. Moreover, alginate, HA, and PEG are still the main biomaterials combined with gelatin. The addition of other component of the ECM such as fibronectin, collagens, or glycosaminoglycans in the hydrogels could enable the production of even more biomimetic platforms. Moreover, gelatin has shown its potential in the creation of vascularized tissues through bioprinting technologies^[^ ^229^ ^]^ that might help in the production of vascularized tumors. Also, in the last years new efforts have been made to study the role of the immune system in the tumor progression.^[^ ^205^ ^]^ The use of microtechnologies such as microfluidic and bioprinting and gelatin hydrogels could lead to potential new 3D in vitro models that takes into consideration the implications of the immune system in cancer.

### 4.3 Fibrin Hydrogels

Fibrin is a natural fibrous protein involved in blood clotting. Fibrin hydrogels can be prepared by fibrinogen polymerization mediated by thrombin, by just combining both components and heating up to 37 °C. Its stiffness can be modulated by manipulating protein concentration, allowing the cell culture of different cell types to fabricate cardiovascular or neural tissues.^[^ ^242^ ^]^ One of the main limitations of such hydrogels is their low transparency, reducing their applicability for 3D HTS applications.^[^ ^243^ ^]^ In an inspired approach, researchers overcame this limitation by adding PEG to the fibrin network, and employed it for manufacturing 3D in vitro models of human lung adenocarcinoma. Lung adenocarcinoma cells (A549) formed 3D spheroids within the hydrogel matrix, and when cocultured with endothelial cells (HUVEC) and normal lung fibroblasts (MRC‐5), microtumors with a more aggressive phenotype were observed. Also, the response to an oncolytic adenovirus therapy on this 3D model resembled better the in vivo response of this therapy than that obtained in 2D cultures.^[^ ^243^ ^]^

**Figure 9:**
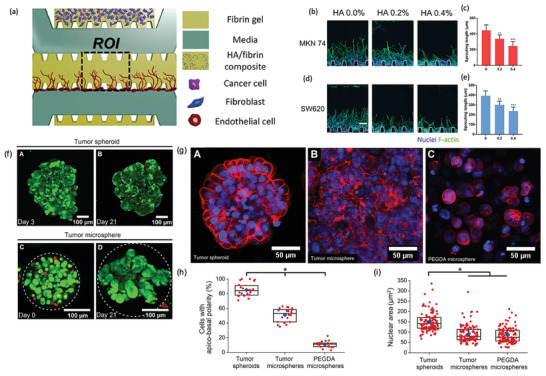
Fibrin hydrogels used for 3D in vitro cancer modeling. a–e) Microfluidic device to recapitulate the bone metastasis from colorectal cancer and gastric cancer and study angiogenesis. The bone TME was recapitulated with the combination of fibrin hydrogels and hydroxyapatite and colon/gastric cancer cells and fibroblasts. a) Schemes of the microfluidic device. b,c) Confocal images of the blood vessels sprouting for b) MKN74 and c) SW620 in hydrogels containing 0.2% and 0.4% of hydroxyapatite (scale bar: 100 µm). Analysis of the sprouting length blood vessels for d) MKN74 and e) SW620 in hydrogels containing 0.2% and 0.4% of hydroxyapatite. Reproduced under the terms of the Creative Commons Attribution license CC‐BY 4.0.^[^ ^245^ ^]^ Copyright 2019, The Authors. f–i) Microtumors of MCF‐7 cells in microspheres of PEG–fibrinogen assembled through a water–oil emulsification. (f‐A to D) Cell viability of a MCF‐7 spheroid and a microtumor. Hydrogels were labeled with calcein AM (green) and ethidium homodimer (red), white lines represents the microparticle (scale bar: 100 µm). g–i) Improved tumorigenicity in microtumor. g) Confocal micrograph of g‐A) tumor spheroids, g‐B) microtumor, and g‐C) PEGDA microspheres (nuclei: blue, actin: red, scale bar: 50 µm). h) reduction in polarity (*n* > 20, *p* < 0.05). i) Reduction in nuclear area (*n* > 100 cells, *p* < 0.05). Blue diamonds, mean; rectangular boxes, lower, medians, and upper quartiles. Cells growing in these microtumors lose the apicobasal polarity, indicating a more malignant phenotype, in comparison to spheroids obtained by a scaffold free approach. Reproduced with permission.^[^ ^246^ ^]^ Copyright 2017, Elsevier.

Fibrin has also been microengineered for obtaining 3D cancer models. Particularly, fibrin hydrogels have been explored as biomaterials for including that were included in microfluidic chips.^[^ ^244^, ^245^ ^]^ For example, a bone metastasis from colorectal cancer and gastric cancer cells model was created using this approach. The bone microenvironment was recapitulated with the combination of fibrin hydrogels and hydroxyapatite,^[^ ^245^ ^]^ and allowed the study of the effect of TME in the advent of bone metastasis and angiogenesis (**Figure** **9**a–c).^[^ ^245^ ^]^ In a recent study, researchers fabricated tumor microspheres of PEG–fibrinogen through a water–oil emulsion method. Cells cultured in this ECM‐mimetic matrix exhibited a higher disorganization within the microgel and lose apicobasal polarity, indicating a more malignant phenotype, when compared to scaffold‐free 3D spheroids.^[^ ^246^ ^]^ Fibrin has also been used as a bioink to 3D bioprint a GBM tumor model, by combination with alginate and genipin, followed by crosslinking with Ca^2+^, thrombin, and chitosan.^[^ ^247^ ^]^ This multi‐component hydrogel enabled U87MG cells to form spheroids with a higher expression of GBM biomarkers and an altered sensitivity to administered drugs when compared with standard 2D models.^[^ ^247^ ^]^ In **Table** **3**, fibrin hydrogels used for 3D in vitro tumor modeling are summarized.

**Table 3:** 3D In vitro cancer models using fibrin and silk fibroin‐based hydrogels. Abbreviations: Collagen I (Col I), gellan gum (GG), glioblastoma multiform (GBM), high throughput screening (HTS), horseradish peroxidase (HRP), human adipose‐derived mesenchymal/stromal stem cells (hAMSC), human umbilical vein cells (HUVEC), polyethylene glycol (PEG), silk fibroin (SF), and tumor microenvironment (TME)

3D in vitro tumor model	Hydrogel composition	Model cells	Application	Achievements	Ref.
Breast cancer	SF + Col I	MDA‐MB‐231	Invasion and migration	The invasion behavior of cancer cells was biphasic. At a fixed Col I concentration, a higher amount of SF increased the stiffness (from 30 to 300 Pa) and reduced hydrogels pore size. The higher invasiveness was observed a 100 Pa.	^[^ ^256^ ^]^
Bone metastasized osteosarcomas	SF + GG	hAMSC/Saos‐2	Recapitulation of bone TME	Hydrogels of 75% GG: 25% SF resembled the osteosarcoma TME and increased osteosarcoma biomarkers.	^[^ ^255^ ^]^
Colon and gastric cancer	Fibrin + hydroxyapatite	SW620/MKN74/HUVEC	Migration and angiogenesis	Microfluidic chip mimicking the bone TME. Higher hydroxyapatite content reduced the migration of cancer cells and the formation of sprouts.	^[^ ^245^ ^]^
Colorectal	SF crosslinked HRP	HCT‐116 cells	Invasion and migration	Microfluidic devices to visualize cell migration in situ. Cells migrated faster and to longer distances in softer hydrogels, and hVCAM‐1 increased the migration distance (which is not observed in Matrigel).	^[^ ^252^ ^]^
GBM	Fibrin + alginate + chitosan	U87MG	Spheroid formation	3D bioprinted hydrogel construct. Cells formed spheroids with a higher expression of GBM markers and a reduced sensitivity to pharmacological treatment.	^[^ ^247^ ^]^
Lung cancer	Fibrin + PEG	A549/HUVEC/MRC5	Drug screening	Cells formed 3D spheroids in the hydrogel matrix, and when cocultured with endothelial cells and fibroblasts exhibited a more aggressive phenotype. Oncolytic adenovirus therapy resembled better the in vivo response in comparison to that obtained with 2D cultures.	^[^ ^243^ ^]^
Others	Fibrin	HUVEC/A549/BxPC3/U‐87 MG/SK‐OV3/HepG2/LoVo/MCF7/LNCaP/786‐O/Lung fibroblasts	HTS	Microfluidic device to study the effect of cancer cells in angiogenesis and the effect of anticancer drugs, compatible with HTS.	^[^ ^244^ ^]^
	Fibrin + PEG	MDA‐MB‐231/SK‐BR‐3/PC‐3/PC‐3‐Met/HT29/MCF‐7	Microtumors fabrication	Microbeads produced by water–oil emulsion method. Cancer cells growing in these microtumors exhibit a more malignant phenotype when compared with 3D spheroids.	^[^ ^246^ ^]^

Interestingly, fibrinogen and thrombin can be isolated from patient's blood, representing a fully human‐based biomaterial that can be used for biomedical applications that can benefit from such features. However, the intrinsically low mechanical properties of fibrin hydrogels and fast degradation has limited its more widespread use for 3D tumor modeling.^[^ ^248^ ^]^ Nonetheless, the addition of other reinforcing materials or tunable concentrations of thrombin/fibrinogen may overcome such limitations to ensure the stability and appropriate mechanical properties of tumors ECM.

### 4.4 Silk Fibroin (SF) Hydrogels

SF is a biocompatible, biodegradable, and noncell‐adhesive protein obtained from silkworm cocoons^[^ ^249^ ^]^ or through genetic engineering.^[^ ^250^ ^]^ Hydrogels can be easily prepared through acidification, increasing ion concentration or temperature, that provoke proteins transition from an amorphous state to more organized *β*‐sheet structures. Alternative solvents to prepare silk‐based hydrogels have been recently proposed, such as ionic liquids.^[^ ^251^ ^]^ Some authors have also reported that hydrogels can be obtained via enzymatic crosslinking^[^ ^252^, ^253^, ^254^ ^]^ mediated by HRP/H_2_O_2_, crosslinking of the tyrosine groups present in the protein (≈5% of SF). The stiffness of these hydrogels can be easily controlled by adjusting the SF concentration. HRP crosslinked hydrogels form transparent gels with protein in a random coil conformation that can be easily injected and supports cancer cell growth. However, after 10 days in culture a transition to a *β*‐sheet conformation is generally observed, a structural shift that is known to promote the apoptosis of cancer cells.^[^ ^253^, ^254^ ^]^ Nevertheless, this limitation can be overcome when the SF is combined with other materials. Gellan gum (GG), an exopolysaccharide similar to glycosaminoglycans presented in the ECM, was combined with SF to recreate bone microenvironments in metastasized osteosarcomas.^[^ ^255^ ^]^ By just blending different ratios of both polymers, spongy‐like hydrogels can be obtained with different mechanical and porosity properties. Hydrogels of 75% GG: 25% SF were able to resemble the osteosarcoma morphology, and an increase in osteosarcoma biomarkers was observed in coculture of human osteosarcoma and hAMSCs. Col I has also been combined with SF modeling breast cancers cell migration.^[^ ^256^ ^]^ Hydrogels with stiffness ranging from 30 to 300 Pa prepared at a fixed Col I concentration and increased SF concentration, showed a biphasic invasion behavior, presenting the higher invasion capacity at stiffness of 100 Pa. This behavior was provoked by a reduction in the porosity of the hydrogel. In Table 3, SF hydrogels used for cancer in vitro modeling are summarized.

The use of SF in tumor modeling is still shyly explored and just started in the last years. A greater understanding of how the conformation changes of the hydrogel occur and their implications in the cancer cell viability should be addressed to ensure its applicability. In addition, the combination with other biomaterials and its chemical modification could better guarantee tumor ECM mimicry. Moreover, the production of these proteins by genetic engineering could also enable the introduction of different biochemical motifs that might avoid the formation of *β*‐sheets.

## 5 Peptide‐Based Hydrogels

To date peptides have been widely used for the functionalization of synthetic and natural polymer hydrogels with sequences for: i) cell adhesion, ii) binding GFs, or iii) enzymatic‐mediated degradation (MMP sequences), etc.^[^ ^257^, ^258^, ^259^ ^]^ Owing to their versatility, peptides can be included as pendant groups in polymer chains^[^ ^260^ ^]^ or used as crosslinkers to form ECM‐mimetic hydrogels.^[^ ^261^ ^]^ In the latter, peptides are generally modified in the terminal groups to incorporate moieties that can react with carboxyl, thiol or amino groups, among others, present in the polymer chains. However, purely peptide‐based hydrogels have been less explored for the fabrication of 3D cancer models. Herein, the following sections will particularly focus on hydrogels entirely comprised by peptides.

In general, peptides used for hydrogels fabrication are amphiphilic molecules that undergo gelation by small changes in pH, ions or temperature, etc.^[^ ^129^ ^]^ These external changes trigger the self‐assembly of the peptides into nanofibers, that lead to the formation of a network comprising inter/intramolecular interactions, physical crosslinking and/or entanglement. The main advantage of using peptide‐hydrogels is that peptides are easily customizable through chemical modification, new amino acids/amino acid sequences can be introduced/removed, or functionalized with new groups, the length of the peptide chain may also be controlled, etc.^[^ ^129^ ^]^ The mechanical properties of peptide hydrogels can be tuned by altering the assembly stimuli or manipulating the peptide sequence or concentration, to generate tailor‐made platforms. The synthetic origin of these materials increases the batch‐to‐batch reproducibility, enables a better control of the physico‐chemical and mechanical properties and a reduction of possible immune‐responses when compared with natural proteins. Nevertheless, the high cost and generally low mechanical properties of peptide hydrogels are still limiting their widespread use.

Herein, the most widely used peptides for generating hydrogel platforms for cancer research are disclosed in the following sections and summarized in **Table** **4**.

**Table 4:** Peptide hydrogels used for 3D in vitro tumor models generation and their main findings. Abbreviations: Basic fibroblast growth factor 2 (FGF‐2), collagen I (Col I), epidermal growth factor (EGF), epithelial mesenchymal transition (EMT), high throughput screening (HTS), matrix metalloproteinases (MMP), peripheral blood mononuclear cells (PBMCs), prostate‐specific antigen (PSA), and vascular endothelial growth factor A (VEGFA)

Peptide hydrogels	3D In vitro tumor model	Achievements	Ref.
RADA16 (Puramatrix) Acn–RADARADARADARADA			
Only RADA16	Ovarian cancer (A2780, A2780/DDP, SK‐OV‐3)	Ovarian cancer cells exhibited a highly proliferative stage and morphological characteristics, similar to Col I hydrogels. Cells displayed a higher resistance to anticancer drugs than in 2D cultures.	^[^ ^265^ ^]^
	Hepatocellular carcinoma (HepG2, SMMC7721)	Cells proliferated in the hydrogel matrix, displayed a spindle‐shaped phenotype, expressed fibronectin and laminin, and produced VEGFA, EGF and FGF2, similarly to Matrigel hydrogels. Cells exhibited lower proliferation and migration in the peptide hydrogels.	^[^ ^266^, ^267^ ^]^
	Breast cancer (MDA‐MB‐231) and nontumor breast cells (MCF10A)	Cancer cells cultured in RADA16 displayed a less malignant phenotype than in Col I or Matrigel. Non‐malignant cells formed acini or 3D spheroids in RADA16 hydrogels supplemented with laminin depending on the stiffness of the hydrogel. Cells exhibited tumor promoting genes upregulation in spheroids form.	^[^ ^268^, ^269^ ^]^
	Lung cancer (A549)	Cells proliferate in culture and formed 3D spheroids, with F‐actin protrusions, exhibited a higher MMP activity that was not correlated to EMT phenotypic changes.	^[^ ^287^ ^]^
RADA16 + polylactic acid/collagen scaffold	Hepatocellular carcinoma (HepG2)	Microfluidic devices to test therapeutics using gradients. Cells formed spheroids into the hydrogel with higher albumin secretion than 3D cultures under static conditions. A life/dead staining showed the cytotoxicity of increasing triton X‐100 concentrations, created with a gradient in the device.	^[^ ^288^ ^]^
RADA16 + Col I + alginate	Diffuse large B cell lymphoma (SUDHL‐10) cultured with fibroblasts (HS‐5 cells) and PBMCs	Microfluidic chip for HTS therapeutic screening in multicellular 3D spheroids. Lenalidomide reduced cancer cells proliferation in 3D spheroids, activated PBMCs and reduced the expression of cytokines associated with a bad prognosis for this type of cancer (IL‐6 and IL‐8, ANG‐1), and increased the secretion of granzyme B.	^[^ ^271^ ^]^
Fmoc‐dipeptides			
Fmoc‐YD and Fmoc‐YK	Hepatome (HepaRG)	3D bioprinted hydrogel cell laden constructs based on a droplet‐based 3D printing. Cancer cells exhibited with high viability post printing and 3D spheroids formation along time in culture.	^[^ ^275^ ^]^
MAX8 VKVKVKVK‐(VDPPT)‐KVEVKVKV‐NH2			
	Medulloblastoma (DAOY and ONS‐76)	Hydrogels were dispensed in well‐plates using a liquid‐handling workstation (HTS compatible approach). Cells formed neurospheres in hydrogel matrix and were more sensitive to anticancer drugs. Cells exhibited a higher expression of nestin and snail 1 neuronal biomarkers.	^[^ ^277^, ^278^ ^]^
H9E FLIVIGSIIGPGGDGPGGD			
	Breast cancer (MCF‐7), cervix cancer (HeLa), liver cancer (HepG2), colon cancer (SW480)	High cell viability, cells tendency to form 3D spheroids in the hydrogel matrix, drugs were able to freely diffuse through the hydrogel matrix, cells cultured in 3D displayed a higher resistance to anticancer drugs than in 2D cultures.	^[^ ^279^, ^280^, ^281^ ^]^
EFK8 EFK8‐I (FEFKFEFK) EFK8‐II (FEFEFKFK)			
	Lung cancer (A549)	Cells formed 3D spheroids or exhibited a more spread morphology depending on hydrogel stiffness or the inclusion of carbon nanotubes.	^[^ ^282^ ^]^
BQ13 AC‐QQKFQFQFEQEQQ‐AM			
	Prostate adenocarcinoma cells (LNCaP)	Cells had a high viability, formed 3D spheroids, similar to those obtained in Matrigel or RADA16, and expressed PSA. Spheroids were more responsive to enzalutamide than in Matrigel and RADA16, inducing a larger PSA reduction.	^[^ ^283^ ^]^

### 5.1 RADA16‐Based Platforms

One of the most used peptide hydrogels are those based in RAD, being R‐arginine, A‐alanine, and d‐aspartic acid. It is based on a yeast protein sequence that forms nanofiber like hydrogels. RADA16 is an amphiphilic and commercially available peptide (Puramatrix) with the sequence AcN–RADARADARADARADA in which gelation is started by the addition of salts such as sucrose.^[^ ^262^ ^]^ These peptide chains form *β*‐sheet that assemble in nanofibers forming a hydrogel network. This biomaterial has been used for different tissue regeneration applications, including cartilage repair, wound healing, etc.^[^ ^263^, ^264^ ^]^

RADA16 has also been tested as a synthetic ECM hydrogel to develop ovarian cancer 3D models.^[^ ^265^ ^]^ When ovarian cancer cells were cultured in RADA16, they exhibited a highly proliferative stage and morphological characteristics, similar to Col I hydrogels, and cells had a higher resistance to anticancer drugs.^[^ ^265^ ^]^ On the other hand, human hepatocellular carcinoma cell cultured in these hydrogels proliferated, exhibited a spindle‐shaped phenotype, while also expressing fibronectin, laminin, and producing VEGFA, epidermal growth factor (EGF), and basic fibroblast growth factor 2 (FGF‐2), similarly to Matrigel hydrogels.^[^ ^266^ ^]^ The only main difference with Matrigel was the lower expression of insulin‐like growth factor (IGF‐1) and Col I,^[^ ^266^ ^]^ suggesting that RADA16‐I could be a good alternative to Matrigel for hepatocellular carcinoma modeling.^[^ ^266^, ^267^ ^]^

On the other side, human breast‐cancer cell line MDA‐MB‐231 cultured in RADA16 platforms displayed a less malignant phenotype than when cultured in Col I or Matrigel.^[^ ^268^ ^]^ In addition, noncancer cells (MCF10A) are able to form normal epithelial acini or tumor‐like spheroids in RADA16 hydrogels laminin‐supplemented depending on the stiffness of the matrix.^[^ ^269^ ^]^ These findings suggest that the suitability of the TME created by RADA16 gels can differ for different cell types. The major limitation of RADA16 hydrogels is their low stiffness, hence the modification of their sequence with amino acid sequence GPGGY57 or blends with other polymers have been used to improve their mechanical properties.^[^ ^270^, ^271^ ^]^ For instance, RADA16 was mixed with alginate to create an HTS compatible model of diffuse large B cell lymphoma.^[^ ^271^ ^]^ The authors developed a microfluidic chip containing 3D spheroids of cancer cells, fibroblasts and lymphocytes that enabled the study of cellular interactions, cell proliferation and lenalidomide cytotoxicity, and allowed the recovery of the soluble factors produced on chip.^[^ ^271^ ^]^ When the artificial tumors were treated with lenalidomide, a reduction in cytokines associated with a bad prognosis for this type of cancer (i.e., IL‐6 and IL‐8), the production of proinflammatory cytokines (i.e., CCL2, CCL3, and CCL4), and angiopoietin‐1 (ANG‐1, associated with highly vascularized tumors), and an increase of the secretion of granzyme B (released by NKs to induce the apoptosis of cancer cells) were all detected.

### 5.2 Aromatic Dipeptide‐Based Platforms

**Figure 10:**
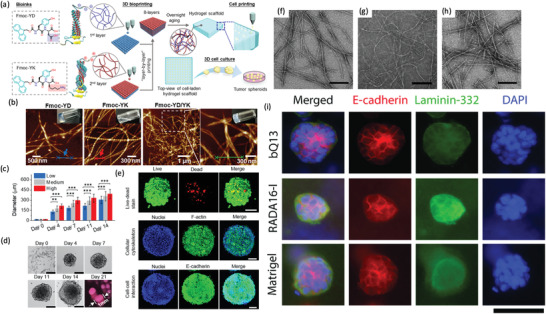
Peptide hydrogels as bioengineered platforms for 3D in vitro cancer modeling. a–e) Fmoc‐dipeptide hydrogels were employed for the fabrication of HepaRG hepatoma 3D spheroids. a) Scheme of fabrication of an Fmoc‐dipeptide hydrogel bioink, bioprinting, and culture of cancer cells to generate 3D spheroids in the macropores of the bioprinted construct. b) Atomic force microscopy micrographs of Fmoc‐YD, Fmoc‐YK, and Fmoc‐YD/YK nanofibers. c) HepaRG hepatoma 3D spheroids growth in Fmoc‐YD/YK hydrogels (^**^ *p* < 0.01 and ^***^ *p* < 0.001. d) Optical photographs of spheroids over time (scale bar: 150 µm). e) Fluoresce microscopy micrographs of 3D spheroids cultured at day 14 (scale bar: 100 µm). First row, live cells (green channel) and dead cells (red channel). Second row, nuclei (blue channel) and F‐actin (green channel) staining. Third row, nuclei (blue channel) and E‐cadherin (green channel) staining. Reproduced with permission.^[^ ^275^ ^]^ Copyright 2019, American Chemical Society. f–i) Use of bQ13 and RADA16‐I for prostate adenocarcinoma cells (LNCaP) 3D spheroids formation. TEM micrographs of negative stained hydrogels of f) bQ13, g) RADA16‐I, and h) Q11 (scale bar = 100 nm). i) Immunofluorescence micrographs of LNCaP spheroids cultured in bQ13, Matrigel, and RADA16‐I. E‐cadherin (red channel), laminin‐332 (secreted ECM, green channel), and cell nuclei (blue channel) (scale bar = 100 µm). Reproduced with permission.^[^ ^283^ ^]^ Copyright 2018, John Wiley and Sons.

Some short peptides bearing a terminal aromatic amino acid have the potential to form hydrogels though the self‐assemble of their chains into nanofiber network through *π*–*π* stacking and H‐bonding interactions.^[^ ^272^ ^]^ FF bearing a 9‐fluorenylmethyloxycarbonyl group (Fmoc‐FF, being F phenylalanine) is the most studied in cell culture applications, because it can self‐assemble under physiological conditions.^[^ ^273^, ^274^ ^]^ Interestingly, it is possible to modify hydrogels stiffness by altering the C‐termini group, the aromatic amino acid, the peptide sequence or its charge. Also, Fmoc‐RGD can be added to the hydrogel to improve cell attachment.^[^ ^273^, ^274^ ^]^ For example, hydrogels can be obtained by mixing two Fmoc‐dipeptides with opposite charge on the terminal residue (Fmoc‐YD and Fmoc‐YK) (**Figure** **10**a–e). This modification improved the mechanical properties and allowed its bioprinting through a droplet‐based 3D printing methodology. The electrostatic interactions between fibers avoided the requirement for an additional crosslinking mechanism. These hydrogels enabled the growth of human hepatoma cells within the 3D printed hydrogel, forming 3D spheroids.^[^ ^275^ ^]^ These spheroids could reach sizes of up to 1–2 mm after 21 days in culture, while expressing key cell–cell adhesion markers (E‐cadherin).

### 5.3 MAX8‐Based Platforms

MAX8 is a 20 amino acid peptide with the sequence VKVKVKVK‐(VDPPT)‐KVEVKVKV‐NH_2_ (being V valine, K lysine, D aspartic acid, P proline, T threonine, and E glutamic acid) with an amphiphilic *β*‐hairpin conformation. It self‐assembles with fast gelation kinetics under physiological conditions into a nanofibrillar network, forming a hydrogel. In low ionic strength and neutral pH solutions, Lysine residues are charged and repel each other, rendering it soluble. However, in cell culture medium the hairpin pairs fold together, hiding the valine and forming nanofibers. It is considered a shear‐thinning solid biomaterial, which means that when shear forces are applied it behaves like a liquid.^[^ ^276^ ^]^ Some of the main advantages is that it can be manipulated at room temperature, and that the stiffness can be tuned from 500 to 10 000 Pa by modulating the peptide sequence, the concentration and the ionic strength of the cell culture medium.^[^ ^129^ ^]^ Cell‐binding sequences can also be installed without altering MAX8 gelling abilities. It is possible to formulate hydrogels in a HTS compatible manner, by dispensing the cell–gel mixtures with a liquid‐handling workstation. Human medulloblastoma cells were cultured in these hydrogels, forming neurospheres more sensitive to cisplatin and vismodegib than in 2D cultures counterparts. It was also observed an increase in the expression of nestin and snail 1, suggesting that it can mimic the TME of medulloblastoma.^[^ ^277^ ^]^ This platform was also employed for automated drug screening, enabling researchers to screen 2202 candidate therapeutic molecules simultaneously.^[^ ^278^ ^]^

### 5.4 Other Peptide Hydrogels

H9e is a peptide with an elastic domain from the spider silk protein and a sequence of the trans‐membrane human muscle l‐type calcium channel, with the sequence FLIVIGSIIGPGGDGPGGD (being F phenylalanine, L leucine, I isoleucine, V valine, G Gly, S serine, D aspartic acid, and P proline). This enables the gel formation under physiological conditions triggered by Ca^2+^. Moreover, this hydrogel has thermosensitivity and shear‐thinning properties. This material has proved its suitability for supporting cancer cells proliferation in 3D.^[^ ^279^, ^280^ ^]^ Breast cancer (MCF‐7), hepatocarcinoma (HepG2), and colon adenocarcinoma (SW480) were included during the crosslinking process, and proliferated overtime leading to the formation of 3D spheroids.^[^ ^279^, ^280^ ^]^ H9e hydrogels exhibited a promising potential for testing anticancer drugs.^[^ ^279^, ^280^, ^281^ ^]^ Cancer cells cultured in these platforms exhibited a dose‐dependent cytotoxicity, and were less susceptible to drugs activity when compared with a standard 2D cell culture.^[^ ^279^, ^280^ ^]^

EFK8 is another peptide that can self‐assemble into 3D hydrogels (F phenylalanine, E glutamic acid, and K lysine). This octapeptide has two variants, EFK8‐I (FEFKFEFK) and EFK8‐II (FEFEFKFK), and both can establish hydrophobic interactions between phenylalanine. Researchers used this peptide to fabricate hydrogels for culturing lung cancer cells, since it exhibits stiffness similar to that of lung tissues.^[^ ^282^ ^]^ Using this approach researchers observed that an increase in the stiffness from 44 to 104 Pa provoked a change in cancer cells morphology, from spheroidal to a stretched one, and a more invasive phenotype. Another peptide used for disease modeling is Q11 (Ac‐QQKFQFQFEQQ‐Am) (where Q is glutamine, K is lysine, F is phenylalanine, and E is glutamic acid), where glutamine residues enable the formation of *β*‐sheet, that aggregate in the form of nanofibers. Their chains can be easily functionalized with short peptides and other groups to improve the mimicry with the native ECM. However, it only remains as liquid at acid pH, limiting its use for encapsulating cells. To overcome this bottleneck researchers modified this peptide to remain liquid at neutral pH, synthetizing bQ13 (Ac‐QQKFQFQFEQEQQ‐Am). Prostate adenocarcinoma cells (LNCaP) had a high viability and proliferate forming 3D spheroids in bQ13 gels (Figure 10f–I), similar to those obtained in Matrigel or RADA16, with higher expression of prostate‐specific antigen (PSA). Cells were also more responsive to enzalutamide than in Matrigel and RADA16, inducing a larger reduction in the PSA levels.^[^ ^283^ ^]^

There are many other peptides that could be interesting platforms for developing 3D in vitro tumor models. For instance, SPG‐178 is another peptide that could undergo hydrogel formation under physiological conditions. This peptide encompasses the following sequence [CH3CONH]‐RLDLRLALRLDLR‐[CONH2], being R‐arginine, l‐leucine, d‐aspartic acid, and A‐alanine. The Leucine amino acid was installed to increase peptide hydrophobicity and allowed it to self‐assemble into a 3D structure that supported cells culture.^[^ ^284^ ^]^

Even though peptide hydrogels exhibit a high versatility and tunability, they have been mainly applied in regenerative medicine and drug delivery applications.^[^ ^285^ ^]^ Nevertheless, the field of peptide synthesis is in continuous development and improved peptide‐based biomaterials are envisioned in the upcoming future. These new advances will make possible not only to recreate the architecture and mechanical properties of the TME, but also, to introduce specific functionalities of the tumor ECM, or to incorporate specific biochemical cues to mediate/activate specific pathways. The knowledge that can arise from these platforms could be endless, as it will allow to infer the effect of specific signals in tumor progression. Moreover, the possibility of controlling the temporal and spatial properties of peptide hydrogels^[^ ^286^ ^]^ due to their self‐assembling nature could lead to a next generation of 4D hydrogels for in vitro cancer modeling.

## 6 Future Perspectives and Concluding Remarks

The development of new biomimetic hydrogels that can recapitulate the complexity of the tumor ECM is fundamental for the development of more physiologically relevant 3D in vitro tumor. These models are envisioned to allow a better understanding of tumor development, to discover new biological targets and to evaluate anticancer drugs bioperformance. Protein and peptide hydrogels have been extensively bioengineered to fabricate these ECM‐biomimetic scaffolds. Collagen and gelatin and their derivatives have been widely used for recreating the TME of different types of cancer, such as breast, GBM, colorectal, and ovarian cancer among other. They have enabled to evaluate the effect of the stiffness, the invasion potential of cancer cells, the establishment of spheroids compatible with HTS, the diffusion of drugs and NPs, the hypoxia and the cross‐talk with stroma or angiogenesis. Proteins can be chemically modified or combined with other biomaterials to tailor hydrogel properties, including stiffness, fiber density, architecture, degradation rate, etc. Moreover, the use of different proteins for recapitulating the TME is still poorly explored. Human‐derived proteins, such as the ones obtained from perinatal tissues^[^ ^289^ ^]^ or from human blood plasma,^[^ ^290^ ^]^ could be a very interesting source of biomaterials to produce humanized structures in cancer models. Moreover, recent advances genetic engineering tools for producing synthetic proteins such as recombinant elastins is another valuable alternative to produce proteinaceous hydrogels with tunable properties that can be valuable for 3D in vitro tumor modeling.^[^ ^291^ ^]^

Apart from full protein‐based biomaterials, synthetic peptides are also opening new avenues in the field of tissue engineering and in vitro disease modeling. The synthetic origin of these biomaterials provides a wide range of reproducible hydrogel properties and the integration of diverse biochemical and biomechanical cues with temporal and spatial control. Despite their huge potential, they are just starting to be explored in the field of cancer modeling. New peptides need to be developed to have more ECM‐biomimetic hydrogels, by including specific functionalities of the tumor ECM.

Even though significant progresses in the development of hydrogel platforms to recapitulate the TME have been accomplished in recent years, there are still numerous challenges that need to be addressed. One of the future directions that should be considered, is that the ECM is constantly changing during tumor progression. Nevertheless, hydrogels properties such as stiffness, fiber density, architecture, composition or nutrients and oxygen permeability are, in general, only studied at the initial state. Indeed, it is important to guarantee that the in vitro model is actually recapitulating the changes occurring in the TME. Therefore, the design of protein/peptide 4D hydrogels that change their biochemical and mechanical properties in the similarly to a native tumor, will enable a deeper understanding of the tumor physiopathology and the discovery of new therapeutic targets. In general, the evaluated parameters include the effect of stiffness, permeability and composition on cancer cells, or in the interaction of stromal and cancer cells. However, there are still important factors that need to be considered and decoupled to decipher biomaterials properties involved in tumor progression, such as the hydrogel architecture, viscoelasticity, spatial variation in the ECM, fibers alignment, etc.

Another challenge that needs to be addressed is models’ predictability/biomimicry. Even though there are diverse protein and peptide‐based models available, none of them can fully mimic the TME, mainly due to the high diversity and heterogeneity of tumors. Moreover, the tumor ECM is a complex network formed by more than 300 components, and the use of one or few protein combinations alone cannot recreate this complexity. A better understanding of the ECM of different tumors will help in the development of new materials that can not only incorporate the most important proteins present in the ECM, but also have the same permeability, architecture, stiffness, etc., than that encountered in native tumor ECM.

Another important point that proteinaceous hydrogel's could also address is the culture of primary cancer cells obtained from patients for “precision cancer medicine.” The high diversity and heterogeneity of tumors leads to a high variability between patient's treatment response, leading to a reduced efficacy in some patient cohorts. Patient‐derived organoids or xenografts can be cultured in hydrogels to model inter‐patient viability and better recapitulate a patient‐specific bioengineered tumor. The screening of different therapeutics in these models is envisioned to enable the selection of the optimal therapeutic and dose for achieving the maximal response for a specific patient, providing a personalized treatment.

Lastly, there are other key aspects that could increase proteinaceous hydrogels use in the development of in vitro models, such as reducing the cost of their fabrication, reproducibility, scalability into HTS, easier manipulation, or mild degradation to harvest the encapsulated cells, which will allow for a more refined analysis of the tumor and stromal cells interactions via omics‐based techniques.

## Conflict of Interest

The authors declare no conflict of interest.
